# Measuring white matter microstructure in 1,457 cannabis users and 1,441 controls: A systematic review of diffusion-weighted MRI studies

**DOI:** 10.3389/fnimg.2023.1129587

**Published:** 2023-03-07

**Authors:** Emily Anne Robinson, John Gleeson, Arush Honnedevasthana Arun, Adam Clemente, Alexandra Gaillard, Maria Gloria Rossetti, Paolo Brambilla, Marcella Bellani, Camilla Crisanti, H. Valerie Curran, Valentina Lorenzetti

**Affiliations:** ^1^Neuroscience of Addiction and Mental Health Program, Healthy Brain and Mind Research Centre, School of Behavioural and Health Sciences, Australian Catholic University, Melbourne, VIC, Australia; ^2^Digital Innovation in Mental Health and Well-Being Program, Healthy Brain and Mind Research Centre, School of Behavioural and Health Sciences, Australian Catholic University, Melbourne, VIC, Australia; ^3^Centre for Mental Health and Brain Sciences, Swinburne University of Technology, Melbourne, VIC, Australia; ^4^Department of Neurosciences and Mental Health, Fondazione IRCCS Ca' Granda Ospedale Maggiore Policlinico, Milan, Italy; ^5^Department of Pathophysiology and Transplantation, University of Milan, Milan, Italy; ^6^Department of Neurosciences, Biomedicine and Movement Sciences, Section of Psychiatry, University of Verona, Verona, Italy; ^7^Clinical Psychopharmacology Unit, University College London, London, United Kingdom

**Keywords:** cannabis, white matter microstructural integrity, diffusion, MRI, dMRI, systematic literature review, neuroimaging, magnetic resonance imaging

## Abstract

**Introduction:**

Cannabis is the most widely used regulated substance by youth and adults. Cannabis use has been associated with psychosocial problems, which have been partly ascribed to neurobiological changes. Emerging evidence to date from diffusion-MRI studies shows that cannabis users compared to controls show poorer integrity of white matter fibre tracts, which structurally connect distinct brain regions to facilitate neural communication. However, the most recent evidence from diffusion-MRI studies thus far has yet to be integrated. Therefore, it is unclear if white matter differences in cannabis users are evident consistently in selected locations, in specific diffusion-MRI metrics, and whether these differences in metrics are associated with cannabis exposure levels.

**Methods:**

We systematically reviewed the results from diffusion-MRI imaging studies that compared white matter differences between cannabis users and controls. We also examined the associations between cannabis exposure and other behavioral variables due to changes in white matter. Our review was pre-registered in PROSPERO (ID: 258250; https://www.crd.york.ac.uk/prospero/).

**Results:**

We identified 30 diffusion-MRI studies including 1,457 cannabis users and 1,441 controls aged 16-to-45 years. All but 6 studies reported group differences in white matter integrity. The most consistent differences between cannabis users and controls were lower fractional anisotropy within the arcuate/superior longitudinal fasciculus (7 studies), and lower fractional anisotropy of the corpus callosum (6 studies) as well as higher mean diffusivity and trace (4 studies). Differences in fractional anisotropy were associated with cannabis use onset (4 studies), especially in the corpus callosum (3 studies).

**Discussion:**

The mechanisms underscoring white matter differences are unclear, and they may include effects of cannabis use onset during youth, neurotoxic effects or neuro adaptations from regular exposure to tetrahydrocannabinol (THC), which exerts its effects by binding to brain receptors, or a neurobiological vulnerability predating the onset of cannabis use. Future multimodal neuroimaging studies, including recently developed advanced diffusion-MRI metrics, can be used to track cannabis users over time and to define with precision when and which region of the brain the white matter changes commence in youth cannabis users, and whether cessation of use recovers white matter differences.

**Systematic review registration:**

www.crd.york.ac.uk/prospero/, identifier: 258250.

## 1. Introduction

Cannabis is the most commonly used regulated substance worldwide, with approximately 209 million users in 2020 alone (UNODC, 2022). Regular cannabis use has been associated with adverse psychosocial outcomes including poorer educational attainment, mental health problems, and cognitive alterations (Volkow et al., 2016; Cookey et al., 2018; Hall et al., 2019; Lorenzetti et al., 2020b; Jansen et al., 2022). The adverse psychosocial outcomes of cannabis use have been partly attributed to aberrant brain integrity in pathways implicated in prominent neuroscientific theories of addiction (Gould, 2010) and high in cannabinoid receptors [e.g., neocortex, hippocampus, thalamus and basal ganglia (Glass et al., 1997)].

Emerging evidence from neuroimaging studies has been used to compare cannabis users and controls, *via* measuring brain integrity *in-vivo* and with millimeter-resolution. This body of work has shown different brain volumetry and function in brain pathways implicated in disinhibition, stress, and reward processing, e.g., orbitofrontal cortex and hippocampus (Harding et al., 2012; Lorenzetti et al., 2016c, 2019; Memedovich et al., 2018; Blest-Hopley et al., 2020; Chye et al., 2021; Sehl et al., 2021; Thomson et al., 2022). However, less is known about whether the white matter pathways between these regions are different between cannabis users and controls. As these white matter tracts are essential for cognition (Filley and Fields, 2016), underlying neural communication, and communication between and within brain regions, it is important to understand how cannabis use affects the integrity of white matter tracts.

Diffusion tensor imaging (DTI) is a tensor-based metric derived from diffusion-MRI, it measures white matter microstructural organization of white matter fibres in the brain (Basser et al., 1994). Recent emerging evidence from DTI studies, shows that cannabis users compared to non-cannabis using controls (henceforth termed controls) have mostly lower Fractional Anisotropy (FA) of white matter fibre tracts in multiple pathways (Bloomfield et al., 2019; Hampton et al., 2019; Chye et al., 2020a; Rossetti et al., 2022). These include: commissural tracts that connect the hemispheres bilaterally (e.g., corpus callosum), tracts connecting frontal regions of the brain (e.g., forceps minor), as well as other association fibres that connect ipsilateral cortical regions. Yet, the group differences were inconsistent, with both higher or lower white matter integrity diffusion-MRI metrics in cannabis users than controls, and lack of significant group differences in some studies (Hampton et al., 2019; Chye et al., 2020a; Rossetti et al., 2022). Such inconsistent findings highlight a lack of consensus regarding white matter changes in cannabis users compared to controls using diffusion-MRI metrics [e.g., FA, Mean Diffusivity (MD), Radial Diffusivity (RD), and Axial Diffusivity (AD)].

Furthermore, emerging literature suggests that white matter integrity in cannabis users is associated with cannabis exposure metrics (e.g., age of cannabis use onset, cannabis dosage, and duration) in a subset of white matter tracts [e.g., corpus callosum and superior longitudinal fasciculus (SLF)] (Hampton et al., 2019; Chye et al., 2020a; Rossetti et al., 2022). However, the nature of such associations were somewhat inconsistent (e.g., location, direction, and the type of diffusion-MRI metric). Therefore, it remains unclear if there are differences in white matter microstructure associated with cannabis exposure and related problems.

Three main limitations of the available synthesis of the diffusion-MRI evidence to date prevent the understanding of the findings regarding white matter integrity changes in cannabis users. First, systematic reviews on the topic published thus far were published 9–12 years ago (Martín-Santos et al., 2010; Baker et al., 2013; Batalla et al., 2013). Therefore, they do not summarize the most recent evidence on the topic and cannot capture the current trends in the evidence and the improved quality of the methodology over time that provide an increasingly fine-grained measure of white matter.

Second, while a review of diffusion-MRI studies of cannabis users was undertaken more recently, it examined general substance using populations, which prevented a detailed summary and discussion of findings in relation to cannabis users specifically (Hampton et al., 2019). Third, other recent syntheses of the literature were not systematic but narrative (Bloomfield et al., 2019; Blest-Hopley et al., 2020; Chye et al., 2020a; Rossetti et al., 2022). Therefore, it is unclear whether cannabis users show systematic differences in the location, direction, and diffusion-MRI metric of white matter microstructural differences.

We aim to overcome the limitations of the literature to date and to systematically integrate the evidence from diffusion-MRI studies of white matter differences between cannabis users vs. controls. In addition, we aim to review the evidence on the associations between diffusion-MRI metrics in cannabis users and levels of cannabis exposure (e.g., age of onset, duration, and dosage), mental health, and other variables.

## 2. Methods

**Figure 1:**
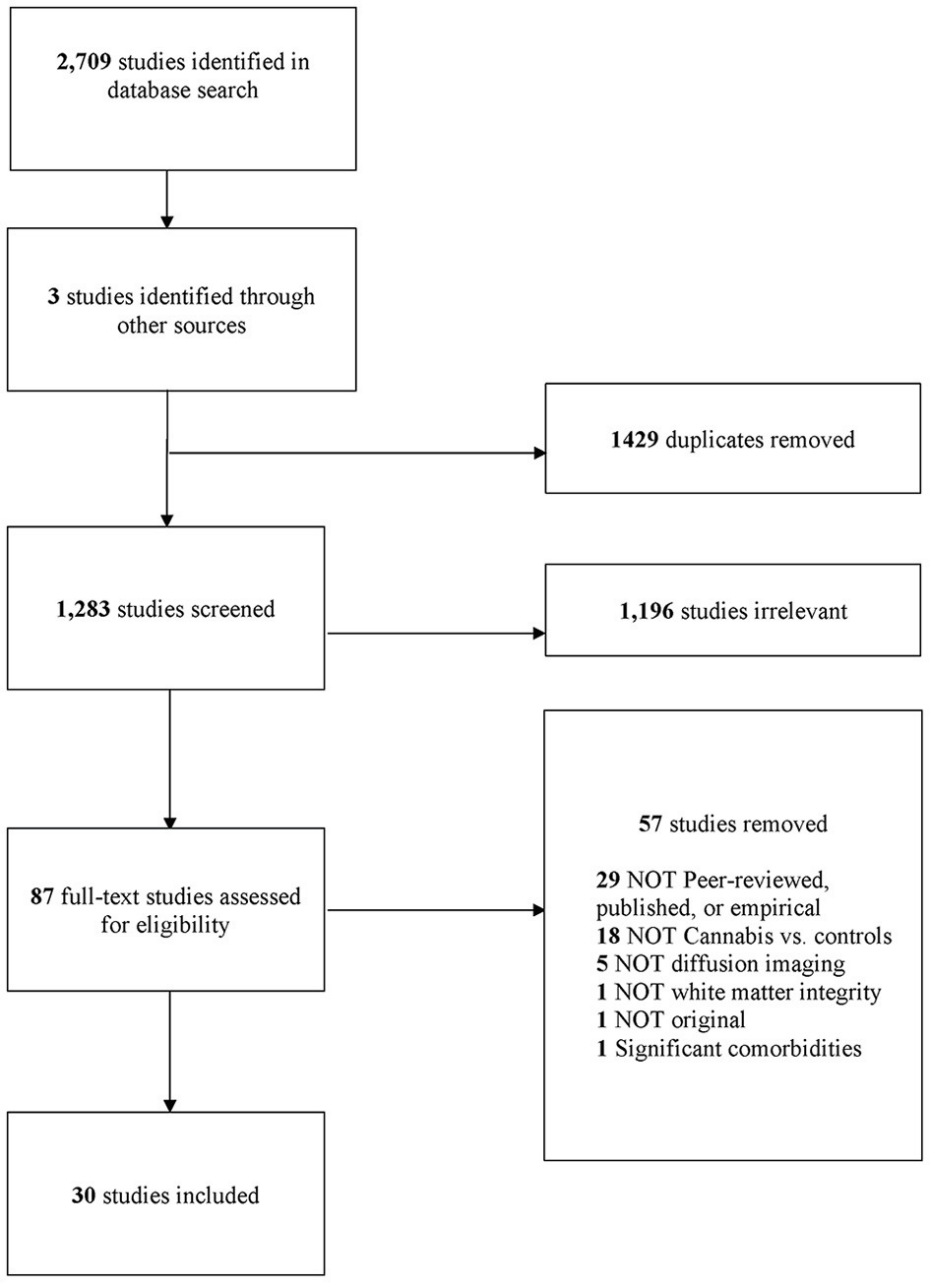
PRISMA diagram (Moher et al., 2009).

This review was preregistered in the International Prospective Register of Systematic Reviews (PROSPERO) (Registration ID: 258250, submitted 08/06/2021, accepted 08/07/21). The systematic literature search, the screening, and the selection of the studies, were reported according to the Preferred Reporting Items for Systematic Reviews and Meta-Analyses (PRISMA) guidelines (Moher et al., 2009), as outlined in Figure 1 and, as per checklist, in Supplementary material 1.

### 2.1. Literature search

A systematic electronic database search was undertaken on the 24 of May 2021 using five databases: MEDLINE, PsycINFO, Web of Science, Embase, and Scopus. The searches encompassed terms related to both “Cannabis” AND “Diffusion-weighted MRI”. Search terms were: (“Diffusion^*^ OR “white matter” OR white-matter OR DW-MRI OR DTI OR DTI-MRI OR dMRI OR “Fractional Anisotropy” OR Tractography OR Connectome OR Connectomics”) AND (Cannabi^*^ OR Marijuana^*^ OR hashish OR marihuana OR kush OR weed). All terms were searched in the title, abstract, keywords, and/or subject headings as appropriate. All study records found in each database were exported into Endnote, and all duplicates were removed. Any additional duplicates found in Covidence were also removed.

The search was rerun on the 7th of December 2022 to identify additional recently published manuscripts.

### 2.2. Inclusion and exclusion criteria

Inclusion criteria were:

i. written in English;
ii. human sample;
iii. use of diffusion-MRI to assess white matter integrity;
iv. compared a cannabis-using group with a control group of persons who do not regularly use cannabis, as defined in each study.

Exclusion criteria were:

i. the sample used illicit substances other than cannabis on a regular basis as defined by each study protocol (e.g., cocaine and methamphetamines);
ii. the sample endorsed lifetime major medical conditions, neurological disorders or mental health disorders (e.g., HIV, Parkinson's disease, and schizophrenia);
iii. the sample was assessed during acute cannabis intoxication;
iv. non-peer reviewed, not published or not empirical studies (e.g., dissertations, conference abstracts, book chapters, case reports, reviews, and meta-analyses);
v. use of neuroimaging techniques other than diffusion-MRI (e.g., PET, functional MRI, and EEG);
vi. outcome measures other than white matter (e.g., gray matter).

### 2.3. Data screening

All studies were screened using the website Covidence (https://www.covidence.org) at both the title/abstract and full text stages. Screening was conducted by ER; any ambiguity in relation to the inclusion of a study was resolved in communication with VL. Studies were first screened against exclusion and inclusion criteria using titles and abstracts. Full-text articles were then further screened for inclusion in the systematic review. Finally, reference lists of (1) studies that met the inclusion criteria for this review and (2) reviews and meta-analyses on similar topics, were examined to identify any further studies that may have been eligible for inclusion in the current review.

### 2.4. Data extraction

Data extraction was conducted by ER and AC. The following information was extracted from tables, figures, and written summaries from each study. These details were summarized into nine tables. Table 1 displays information on publication characteristics (e.g., first author and year of publication); sample characteristics (e.g., sample size, sex, and age); and cannabis use levels (e.g., age of cannabis use onset and abstinence period). Table 2 outlines key definitions for technical terms regarding diffusion-MRI metrics and analyses used throughout the paper.

**Table 1:** Overview of mean (standard deviation) of studies' sample sizes, sex composition, age, and cannabis exposure metrics.

**References**		**N (female)**		**Age, yrs**		**Cannabis use levels**					
		**Cannabis**	**Control**	**Cannabis**	**Control**	**Duration, yrs**	**Age of onset, yrs**		**Dosage, cones/mo**	**Frequency**	
							**Regular Use**	**1st Use**		**Days/mo**	**Occasions**
Cousijn et al. (2022)		39 (17)	28 (16)	21.5 (2.3)	21.4 (2.0)	4.1 (2.2)	15.3 (1.90)	_	239.2 (278.2)	20.4 (7.4)	_
Knodt et al. (2022)		82 (29)	192 (113)	~45	~45	_	_	_	_	_	_
Lichenstein et al. (2022)	*Moderate-BL*	52 (0)	53 (0)	~20	~20	_	_	16.1 (2.1)^*^	_	4.0 (5.2)	_
	*FU*			~22	~22	3.8 (2.0)	_		_	3.4 (4.5)	_
	*Heavy-BL*	53 (0)		~20	~20	_	_	14.7 (1.8)^*^	_	22.4 (10.7)	_
	*FU*			~22	~22	6.6 (1.6)	_		_	18.9 (11.8)	_
Koenis et al. (2021)		42 (21)	110 (60)	38 (19–69)	40 (19–69)	11 (1–35)	_	_	_	_	_
Manza et al. (2020)		89 (25)	89 (25)	28.6 (3.9)	28.6 (3.5)	_	_	_	_	_	_
Sweigert et al. (2020)		26 (13)	25 (12)	26.2 (4.1)	26.4 (5.1)	4.1 (3.3)		17.4 (4.6)	135.1 (117.2)		Monthly or less *[n = 0]* 2–4x per mo *[n = 5]* 2–3x per wk *[n = 8]* >4x per wk *[n = 13]*
Levar et al. (2018)		19 (11)	22 (12)	20.6 (2.5)	21.6 (1.9)	4.4 (1.7)	_	16.2 (1.7)	76.5 (65.9)	11.7 (6.4)	_
Jakabek et al. (2016)		56 (32)	20 (12)	32.3 (10.1)	30 (10.6)	15.5 (9.7)	16.3 (2.6)	15.1 (2.3)	460.7 (350.1)	25.5 (8.0)	_
Orr et al. (2016)		465 (244)	394 (237)	28.8 (3.7)	28.8 (3.7)	_	_	≤ 14 yrs *[n = 52]* 15–17 yrs *[n = 170]* 18–20 yrs *[n = 151] ≥21 yrs [n = 93]*	_	_	*lifetime occ:* 1–5 *[n = 174]* 6–10 *[n = 63]* 11–100 *[n = 94]* 101–999 *[n = 60]* 1,000+ *[n = 75)*
Rigucci et al. (2016)	Occasional	11	22	_	_	7.2 (5)	_	<15 yrs *[n = 6]*	_	_	_
	Daily use	11						>15 yrs *[n = 16]*			
Yucel et al. (2016)		74 (34)	37 (19)	32.7 (10.8)	30.0 (11.3)	15.67 (9.7)	17.0 (3.5)	_	414.0 (303.6) *last yr*	24.6 (8.9) *last yr*	_
Becker et al. (2015)	*BL*	23 (7)	23 (7)	19.5 (0.7)	19.2 (2.3)	_	15.4 (1.2)	_	3,032.6 (2,395.3) *hits/last yr*	_	11.2 (13.8) *max hit/past yr*
	*FU*			21.8 (0.8)	21.3 (2.4)	_	_	_	2,637.9 (2,203.8) *hits/last yr*	_	16.2 (28.7) *max hit/past yr*
Epstein and Kumra (2015)	*BL*	19 (8)	29 (16)	17.9 (1.50)	16.5 (2.20)	_	_	_	_	712 (399) *life*	_
	*FU*									348 (270) *day interscan*	
Shollenbarger et al. (2015)		33 (12)	34 (20)	21.2 (18–25)	21.2 (18–25)	_	17.9 (10–24)	_	137.09 (6.5–973.7)	_	_
Epstein et al. (2014)		31 (9)	55 (28)	17.9 (2.4)	16.5 (2.6)	_	<17		_	_	_
Filbey et al. (2014)		48 (15)	62 (23)	17.9 (8.3)	28.3 (8.3)	9.8 (8.0)	_	18.1 (3.4)^*^	_	_	48.1 (6.1)
Gruber et al. (2014)	*Early onset*	25 (7)	18 (11)	17.9 (5.9)	23.1 (3.5)	8.8 (5.7)	14.5 (0.7)	_	763.9 (989.2)	_	81.5 (40.8)
	*Late onset*					5.1 (4.4)	17.9 (2.1)	_	347.2 (289.9)	_	67.4 (31.2)
Jacobus et al. (2013a)	*BL*	21 (8)	16 (8)	17.9 (16–19)	17.9 (16–19)	_	_	_	_	_	_
	*FU-18 mo*			19.4 (17–20)	19.4 (17–20)	_	_	_	_	_	_
	*FU-27 mo*			20.9 (19–21)	20.9 (19–22)	_	_	_	_	_	_
Jacobus et al. (2013b)	*BL*	47 (19)	49 (13)	18 (0.9)	17.6 (0.8)	_	_	_	_	_	471.0 (357.1) *life*
	*FU*			19.5 (0.9)	19 (0.9)	_	_	_	_	14.8 (15.8) *interscan*	
Zalesky et al. (2012)		59 (31)	33 (19)	33.4 (10.9)	31.5 (12.0)	15.6 (9.5)	16.7 (3.3)	_	441 (426)	25.7 (8.1)	_
Gruber et al. (2011)		15 (1)	15 (1)	17.9 (8.7)	25.2 (8.4)	10.1 (9.7)	14.9 (2.5)	_	332.4 (27.8)	_	_
Kim et al. (2011)		12 (0)	13 (0)	19.3 (1.0)	21 (3.8)	3.36 (2.5)		16 (2.4)	_	_	21.7 (7.4)
Yücel et al. (2010)		11 (4)	8 (6)	19.4 (1.9)	19.7 (2.7)	_	_	15 (1.6)^*^	292.2 (182.6)	_	_
Ashtari et al. (2009)		14 (0)	14 (0)	19.3 (0.8)	18.5 (1.4)	5.3 (2.1)	_	13.1 (1.6)^*^	529.6 (237.4)	_	_
Bava et al. (2009, 2010)^#^		36 (10)	36 (10)	17.9 (0.9)	17.8 (0.8)	_	14.7 (3.1)	13.9 (2.0)	_	11.6 (8.4)	_
Jacobus et al. (2009)		14 (2)	14 (2)	18.2 (0.7)	17.3 (0.8)	_	_	_	_	_	51.0 (54.1)
Arnone et al. (2008)		11 (0)	11 (0)	25.0 (3.0)	23.4 (2.9)	9.0 (3.5)	_	15.27 (2.8)	_	_	_
Delisi et al. (2006)		10 (1)	10 (1)	17.9 (2.9)	23 (4.4)	_	_	< 18	_	_	_
Gruber and Yurgelun-Todd (2005)		9 (1)	9 (1)	17.9 (3.6)	26.2 (3.1)	_	_	14.1^*^	513.6	_	_

Units are as indicated unless otherwise noted. mo, months; BL, baseline; FU, follow up; occ, occasions; wk, weeks; yrs, years; yr, year. For cannabis exposure metrics, mean and standard deviation was reported, where available, unless otherwise stated. Range was reported (where available) where standard deviation was not measured.

* Unspecified if age of first cannabis use onset or age of regular cannabis use onset.

# Participants were the same across studies.

**Table 2:** Description of (1) diffusion-MRI metrics and (2) other novel metrics of white-matter integrity used in the reviewed studies.

**Diffusion MRI metric/methods**	**Acronym**	**Description**	**Interpretation of low white matter integrity**
**Diffusion tensor imaging metrics**			
Fractional anisotropy	FA	The directionality and coherence of water diffusivity within white matter fibre tracts as a number from 0 (directional and isotropic diffusion) to 1 (random or anisotropic diffusion) (Basser et al., 1994).	Lower scores.
Mean diffusivity/apparent diffusion coefficient/trace	MD^*^ ADC	The total amount of water diffusivity in a voxel, which is related to the amount of water in the extracellular space (Basser, 1995; Pierpaoli et al., 1996).	Higher scores.
Radial diffusivity	RD	Water diffusivity perpendicular to white matter tracts (Basser, 1995; Song et al., 2002).	Higher scores.
Axial diffusivity	AD	Water diffusivity parallel to white matter tracts—best measured in regions of coherently orient axons with no fibre crossings (Basser, 1995; Song et al., 2002).	Lower scores.
**Other metrics**			
Normalized characteristic path length	-	Characteristic path length of the whole brain network, normalized to appropriate null network (Bullmore and Sporns, 2009).	Higher scores.
Small worldness	-	Capacity of a network for an energy-efficient balance between network segregation and network integration segregation, relative to an appropriate random network (Bullmore and Sporns, 2009).	Lower scores.
Local efficiency	-	The global efficiency (i.e., average inverse shortest path length between all pairs of nodes in the network) computed on the node's neighbors (Bullmore and Sporns, 2009).	Lower scores.
Network matrix	-	The number of connections and mean weights of the global network (Kim et al., 2011).	Lower scores.
Network based statistic	-	Novel metric for identifying network connectivity differences using non-parametric multiple comparisons (Zalesky et al., 2012).	Lower scores.
Fibre bundle length	-	The mean values from all voxels across the tract, which are then used to determine the length of the fibre bundle (Levar et al., 2018).	Lower scores.
**Methods**			
Voxel-based analysis	VBA	Analysis on entire voxel grid within the brain or regions of interest within the brain (Abe et al., 2010).	-
Tractography	-	Local fibre orientations delineated to create inferred pathways connecting distant regions of the brain—allows for analysis on specific white matter pathways (Mori and Van Zijl, 2002).	-
Tract-based spatial statistics	TBSS	Analysis only on voxels on a mean (template) skeleton representative of a white matter pathway (Smith et al., 2006).	-
Tracts constrained by underlying anatomy	TRACULA	Analysis on probabilistic reconstruction of major white matter pathways by utilizing prior information on the anatomy of pathways from a set of training subjects (Yendiki et al., 2011).	-

* MD, ADC, and trace are often interchangeable in the literature, for the purpose of this review, all of these metrics are labeled as MD for clarity purposes.

Table 3 includes an overall summary of differences in white matter integrity per diffusion-MRI metric in cannabis users compared to non-using controls. In Tables 4–7 we also include information about: the location, significance, and direction of group differences in white matter integrity, and their association with the level of cannabis use, psychopathology symptom scores, cognitive performance, and other variables. We summarized results from group differences and correlations as a function of the examined diffusion-MRI metrics examined. The diffusion-MRI metrics included (1) FA in Table 4; (2) MD, Trace, and apparent diffusion coefficient (ADC) in Table 5; (3) RD in Table 6 and; (4) AD in Table 7. Table 8 overviews results from associations between white matter integrity and levels of cannabis exposure and other variables. Finally, Table 9 contains information relating to studies with longitudinal findings, including follow-up time, group differences, and associations with levels of cannabis use and other key variables.

**Table 3:** Summary of differences in white matter integrity per diffusion-MRI metric in cannabis users compared to controls.

**White matter tract**	**FA**		**MD**		**RD**		**AD**		**FBL**	
	↓	↑	↓	↑	↓	↑	↓	↑		↑
Superior longitudinal fasciculus	7	-	-	1	-	-	-	-	-	-
Corpus callosum	5	1	-	4	1	1	-	-	-	-
Anterior thalamic radiation	2	1	-	1	1	1	-	-	-	-
Internal capsule	3	1	-	1	-	1	-	-	-	-
Uncinate fasciculus	3	-	1	-	1	-	-	-	-	1
Inferior frontal-occipital fasciculus	3	-	-	-	1	-	-	-	-	-
Forceps major	1	-	-	-	-	-	-	-	-	-
External capsule	1	-	-	-	-	-	-	-	-	-
Corona radiata	3	-	-	-	-	-	-	-	-	-
Posterior thalamic radiation	1	-	-	-	-	-	-	-	-	-
Forceps minor	1	1	-	1	1	-	-	-	-	-
Frontal region^*^	2	-	-	-	-	-	-	-	-	-
Temporal gyrus^*^	3	-	-	1	-	2	-	-	-	-
Adjacent to the hippocampus	1	-	-	-	-	-	-	-	-	-
Arcuate fasciculus	1	-	-	-	-	1	-	-	-	-
Crus cerebri^*^	1	-	-	-	-	-	-	-	-	-
Temporo-thalamic^*^	1	-	-	-	-	-	-	-	-	-
Occipito-frontal^*^	1	-	-	-	-	-	-	-	-	-
Middle cerebellar peduncle	1	1	1	-	1	-	-	-	-	-
Occipito cuneus^*^	-	1	-	-	-	-	-	-	-	-
Inferior longitudinal fasciculus	-	-	1	-	-	-	-	-	-	-
Middle frontal gyrus^*^	-	-	1	-	-	-	-	-	-	-
Posterior cingulate^*^	-	-	1	-	-	-	-	-	-	-
Occipito-lingual gyrus^*^	-	1	-	-	-	-	-	-	-	-
Anterior cingulate cortex^*^	-	-	-	1	-	-	-	-	-	-
Motor tracts^*^	-	-	-	-	-	-	1	-	-	-

-, no findings;

* listed gray matter region in results; suggests white matter tracts within region, FA, fractional anisotropy; MD, mean diffusivity; RD, radial diffusivity; AD, axial diffusivity; FBL, fibre bundle length; ↓, Cannabis users < Controls; ↑, Cannabis users > Controls. In addition, the network related metrics (e.g., small worldness) are not represented in this table as they relate to the network as a whole and not white matter tracts.

**Table 4:** Overview of *fractional anisotropy* differences in cannabis users compared to controls, and their association with cannabis exposure levels and other variables.

**References**	**Cannabis users vs. Controls**	**Correlations**
Cousijn et al. (2022)	n.s.	pos. cor. onset age and inferior longitudinal fasciculus and uncinate fasciculus. n.s. duration, dosage, dependence scores
Knodt et al. (2022)	*n.s. [global average] n.s. [tractwise]*	*Subgroup analysis* Global average in more persistent regular users < less persistent regular users. n.s. persistent dependence *[global average]*. n.s. persistent regular use, persistent dependence *[tractwise]*
Lichenstein et al. (2022)	↑ Anterior thalamic radiations *[moderate use vs. low/no use]*	n.s. onset age, duration, and frequency of use. *Subgroup analysis*. Cingulum moderate use > heavy use. Anterior thalamic radiations moderate use > heavy use
Koenis et al. (2021)	↓ SLF, anterior thalamic radiations, forceps *major*, inferior fronto-occipital fasciculus	-
Manza et al. (2020)	↓ SLF, uncinate fasciculus, ext. capsule, corpus callosum (splenium), corona radiata (sup., post.), temporal (inf.), thalamic radiation (post.)	-
Sweigert et al. (2020)	↑ Middle cerebellar peduncle	pos. cor. craving scores (MCQ-SF) and middle cerebellar peduncle *trend*. n.s. CUDIT-R total score
Levar et al. (2018)	n.s. uncinate fasciculus	-
Jakabek et al. (2016)	↓ Forceps *minor [TRACULA]*. n.s. *[TBSS]*	pos. cor. dosage and cingulate gyrus *[TRACULA]* pos. cor. dosage and forceps *minor* neg. cor. dosage and anterior thalamic radiation *[tractography]* n.s. frequency of current use neg. cor. duration and inferior longitudinal fasciculus [TBSS]
Orr et al. (2016)	n.s.	pos. cor. onset age and SLF, inferior longitudinal fasciculus, lateral prefrontal cortex, corpus callosum (ant./post.), forceps *minor/major*
Rigucci et al. (2016)	n.s. corpus callosum	n.s.
Yucel et al. (2016)	n.s. adjacent the hippocampus	-
Becker et al. (2015)	↑ Corpus callosum (genu)	-
Epstein and Kumra (2015)	n.s. inferior longitudinal fasciculus, inferior fronto-occipital fasciculus, corticospinal tract	-
Shollenbarger et al. (2015)	↓ Uncinate fasciculus	neg. cor. depression symptoms and anterior thalamic radiation, uncinate fasciculus neg cor. apathy symptoms and uncinate fasciculus
Epstein et al. (2014)	↓ Inferior fronto-occipital fasciculus	-
Filbey et al. (2014)	↑ Forceps *minor* n.s. forceps *major*	Quadratic assoc. duration and forceps *minor* (larger with initial regular use and lower with continued use)
Gruber et al. (2014)	↓ Corpus callosum (genu), internal capsule *trend ↓* internal and external capsules	pos. cor. onset age and corpus callosum (genu) neg. cor. BIS (attention, motor) and corpus callosum (genu) *trend* neg. cor. BIS (total) and corpus callosum (genu)
Jacobus et al. (2013a)	↓ Corpus callosum (splenium, genu), inferior fronto-occipital fasciculus, anterior thalamic radiations, uncinate fasciculus, SLF, internal capsule (ant./post. limb), corona radiata (ant., sup.)	n.s. global cognitive performance
Jacobus et al. (2013b)	n.s. fornix, superior corona radiata, superior fronto-occipital fasciculus, SLF	-
Gruber et al. (2011)	↓ Frontal region *trend ↓* corpus callosum (genu)	pos. cor. onset age and frontal region and corpus callosum (genu) pos. cor. BIS (total, motor) and frontal region. pos. cor. BIS (total, attention) and frontal region neg. cor. duration and corpus callosum (genu)
Yücel et al. (2010)	↓ Tracts adjacent to the hippocampus, SLF	n.s. onset age, duration, dosage
Ashtari et al. (2009)	↓ Internal capsule (post.), thalamic radiation, mid./sup. temporal gyrus *[voxel wise analysis covariance]* ↓ Arcuate fasciculus*[tractography] trend ↓* arcuate tract	n.s. onset age, duration, dosage, abstinence length
Bava et al. (2009, 2010)	↑ SLF (arcuate), occipital—cuneus, internal capsule (ant. limbic) ↓ SLF, corpus callosum (splenium), inferior longitudinal fasciculus, crus cerebri, postcentral/ superior temporal/and inferior frontal gyri (opercular/insular), temporo-thalamic and occipito-frontal tracts	pos. cor. freq. (days/month) and SLF pos. cor. lifetime cannabis use and occipito-frontal tract
Jacobus et al. (2009)	↓ Superior corona radiata, SLF, middle cerebellar peduncle	pos. cor. lifetime cannabis hits and left superior corona radiata clusters pos. cor. cannabis hits past 3 months) and SLF
Arnone et al. (2008)	n.s. corpus callosum	n.s. onset age, duration and corpus callosum subregions
Delisi et al. (2006)	↑ ACC, medial frontal, cingulate and superior gyrus, precentral, parietal (inf.)	-
Gruber and Yurgelun-Todd (2005)	n.s. corpus callosum (genu and splenium), ACC	-

ACC, anterior cingulate cortex; Ant, anterior; Assoc, associated; BIS, Barratt Impulsiveness Scale; Cor, correlated; Ext., external; Inf, inferior; n.s., non-significant; Neg, negative; Pos, positive; Post, posterior; SLF, superior longitudinal fasciculus; Sup, superior; TBSS, tract-based spatial statistics; TRACULA, TRActs Constrained by UnderLying Anatomy; ↓, Cannabis Users < Controls; ↑, Cannabis Users > Controls.

**Table 5:** Overview of *mean diffusivity* differences in cannabis users compared to controls, and their association with cannabis exposure levels and other variables.

**References**	**Cannabis users vs. Controls**	**Correlations**
Cousijn et al. (2022)	n.s.	n.s. onset age, duration, grams/past 2 weeks, dependence severity
Lichenstein et al. (2022)	n.s. anterior thalamic radiations, cingulum	-
Sweigert et al. (2020)	↓ Middle cerebellar peduncle	neg. cor. craving scores (MCQ-SF) and middle cerebellar peduncle n.s. CUDIT-R total score
Levar et al. (2018)	n.s. uncinate fasciculus	-
Orr et al. (2016)	n.s.	-
Rigucci et al. (2016)	↑ Corpus callosum	*Subgroup analysis*: daily use > occasional use > controls daily/high potency use > low potency use > controls and weekly use early > late onset (< 15 vs. >15 years) *trend*
Shollenbarger et al. (2015)	↑ Forceps *minor*, uncinate fasciculus *trend* ↑ anterior thalamic radiations	pos. cor. depression symptoms and anterior thalamic radiations
Filbey et al. (2014)	n.s. forceps *major* and *minor*	-
Gruber et al. (2014)	↑ corpus callosum (genu)	-
Gruber et al. (2011)	↑ Corpus callosum (genu)	neg. cor. onset age and frontal region, corpus callosum (genu) pos. cor. duration and corpus callosum (genu)
Ashtari et al. (2009)	↑ mid./sup. temporal gyrus tracts, internal capsule, thalamic radiation *[voxel wise analysis of covariance]* ↑ arcuate *[tractography]*	n.s. onset age, duration, amount of use, and length abstinent
Bava et al. (2009, 2010)	↑ Occipital –lingual gyrus tracts ↓ Inferior longitudinal fasciculus	trends neg. cor. hits/month and inferior longitudinal fasciculus *trend*
Jacobus et al. (2009)	n.s.	-
Arnone et al. (2008)	↑ Corpus callosum (prefrontal cortex subregion)	pos. cor. duration and corpus callosum (prefrontal cortex subregion) *trend* n.s. onset age and corpus callosum
Delisi et al. (2006)	↓ Middle frontal gyrus, posterior cingulate	-
Gruber and Yurgelun-Todd (2005)	*trend ↑* ACC, corpus callosum (genu, splenium)	-

ACC, anterior cingulate cortex; Cor, correlated; Mid, middle; n.s., non-significant; Neg, negative; Pos, positive; Sup, superior; ↓, Cannabis Users < Controls; ↑ Cannabis Users > Controls.

**Table 6:** Overview *of radial diffusivity* differences in cannabis users compared to controls, and their association with cannabis exposure levels and other variables.

**References**	**Cannabis users vs. controls**	**Correlations**
Cousijn et al. (2022)	n.s.	n.s. onset age, duration, grams/past 2 wks and dependence severity
Lichenstein et al. (2022)	n.s. anterior thalamic radiations, cingulum	-
Sweigert et al. (2020)	↓ Middle cerebellar peduncle	Neg. cor craving scores (MCQ-SF) and middle cerebellar peduncle n.s. CUDIT-R total score
Levar et al. (2018)	n.s. uncinate fasciculus	-
Jakabek et al. (2016)	n.s.	Neg. cor. duration and angular bundle n.s. frequency of current use
Orr et al. (2016)	n.s.	Neg. cor. onset age and SLF, inferior longitudinal fasciculus, lateral prefrontal cortex, corpus callosum (anterior/posterior)
Rigucci et al. (2016)	*trend ↑* corpus callosum	Corpus callosum daily/high potency users > low potency users > never used/used weekly
Becker et al. (2015)	*trend ↓* corpus callosum (genu)	-
Filbey et al. (2014)	↓ forceps *minor*	Quadratic assoc. duration and forceps *minor* (larger with initial regular use and lower with continued use)
Zalesky et al. (2012)	n.s.	Pos. cor. onset age and commissural fibres, fimbria
Ashtari et al. (2009)	↑ middle temporal gyrus tracts, superior temporal gyrus, internal capsule, thalamic radiation [v*oxel wise analysis of covariance]* ↑ arcuate fasciculus [*tractography]*	n.s. onset age, duration, dosage, abstinence length

Cor, correlated; n.s., non-significant; neg, negative; pos, positive; SLF, superior longitudinal fasciculus; wks, weeks; ↓, Cannabis Users < Controls; ↑ Cannabis Users > Controls.

**Table 7:** Overview of *axial diffusivity* differences in cannabis users compared to controls, and their association with cannabis exposure levels and other variables.

**References**	**Cannabis users vs. controls**	**Correlations**
Cousijn et al. (2022)	n.s.	n.s. onset age, duration, grams/past 2 wks, dependence severity
Lichenstein et al. (2022)	n.s. anterior thalamic radiations, cingulum	-
Sweigert et al. (2020)	n.s. inferior, superior, and middle cerebellar peduncles, pontine crossing tract	Neg. cor craving scores (MCQ-SF) and middle cerebellar peduncle n.s. CUDIT-R total score
Levar et al. (2018)	n.s. uncinate fasciculus	-
Jakabek et al. (2016)	n.s.	Pos. cor. duration and cingulate gyrus. Neg. cor. onset age and cingulate gyrus. n.s. frequency of current use
Orr et al. (2016)	n.s.	-
Rigucci et al. (2016)	↑ corpus callosum	*Subgroup analyses:* corpus callosum in daily users > occasional users > controls in corpus callosum in daily and high potency users > low potency users > controls and weekly users *trend* corpus callosum in age onset < 15 years vs. >15 year
Filbey et al. (2014)	n.s.	-
Zalesky et al. (2012)	n.s.	Pos. cor. onset age and commissural fibre, fimbria
Ashtari et al. (2009)	↓ superior temporal gyrus tracts and internal capsule *[voxel wise analysis of covariance]* ↓ motor tracts *[tractography]*	n.s. onset age, duration, dosage, abstinence length

Cor, correlated; n.s., non-significant; Neg, negative; Pos, positive; wks, weeks; ↓, Cannabis Users < Controls; ↑ Cannabis Users > Controls.

**Table 8:** Summary of significant correlations found between measures of white matter integrity and indices of cannabis use, cognition, alcohol use, and mental health where findings were investigated in 4 or more studies.

	**FA**																		**MD**						**RD**									**AD**				**Other**
	↓					↑													↓				↑		↓							↑		↓		↑		↑
	**CC**	**ILF**	**FMi**	**ATR**	**UF**	**CC**	**ILF**	**UF**	**LP**	**FR**	**CG**	**SLF**	**PFC**	**FMi**	**FMa**	**OF**	**SCR**	**MCP**	**ILF**	**CC**	**FR**	**MCP**	**CC**	**ATR**	**SLF**	**CC**	**MCP**	**ILF**	**LP**	**AB**	**FMi**	**CF**	**FIM**	**CG**	**MCP**	**CF**	**CG**	**GE**
Age of onset	-	-	-	-	-	4	2	1	1	-	-	1	1	1	1	-	-	-	-	1	1	-	-	-	1	1	-	1	1	-	-	1	1	1	-	1	-	-
Duration	1	1	1	-	-	-	-	-	-	-	-	1	-	-	-	1	-	-	-	1	-	-	2	-	-	-	-	-	-	1	1	-	-	-	-	-	1	-
Dosage	-	-	-	1	-	-	-	-	-	-	1	1	-	1	-	-	1	-	1	-	-	-	-	-	-	-	-	-	-	-	-	-	-	-	-	-	-	-
Frequency	-	-	-	-	-	-	-	-	-	-	-	1	-	-	-	-	1	-	-	-	-	-	-	-	-	-	-	-	-	-	-	-	-	-	-	-	-	-
Impulsivity	2	-	-	-	-	1	-	-	-	2	-	-	-	-	-	-	-	-	-	-	-	-	-	-	-	-	-	-	-	-	-	-	-	-	-	-	-	-
Neurocognition	-	-	-	-	-	-	-	-	-	-	-	-	-	-	-	-	-	-	-	-	-	-	-	-	-	-	-	-	-	-	-	-	-	-	-	-	-	-
Memory	-	-	-	-	-	-	-	-	-	-	-	-	-	-	-	-	-	-	-	-	-	-	-	-	-	-	-	-	-	-	-	-	-	-	-	-	-	-
Alcohol use	-	-	-	-	-	-	-	-	-	-	-	-	-	–	-	-	-	-	-	-	-	-	-	-	-	-	-	-	-	-	-	-	-	-	-	-	-	1
Mental health	-	-		1	2	-	-	-	-	-	-	-	-	-	-	-	-	-	–	-	-	-	-	1	-	-	-	-	-	-	-	-	-	-	-	-	-	-
Craving	-	-		-	-	-	-	-	-	-	-	-	-	-	-	-	-	1	-	-	-	1	-	-	-	-	1	-	-	-	-	-	-	-	1	-	-	-

↓, negative correlation; ↑, positive correlation; -, not examined; AD, axial diffusivity; FA, fractional anisotropy; MD, mean diffusivity; RD, radial diffusivity. White Matter Tracts: CC, corpus callosum; CG, cingulate gyrus, listed grey matter region in results, suggests white matter tracts within region; ATR, anterior thalamic radiations; FMi, forceps minor; FMa, forceps major; FR, frontal region, listed grey matter region in results, suggests white matter tracts within region; OF, occipito-frontal; SCR, superior corona radiata clusters; FIM, fimbria; AB, angular bundle; CF, commissural fibres; GE, global efficiency, graph theory metric, not a white matter tract; SLF, superior longitudinal fasciculus; ILF, inferior longitudinal fasciculus; UF, uncinate fasciculus; LP, lateral prefrontal cortex, listed grey matter region in results, suggests white matter tracts within region; AB, angular bundle.

**Table 9:** Overview of the effects of time, and group-by-time effects on white matter, in longitudinal studies of cannabis users and controls, and their association with cannabis exposure levels and other variables.

**References**	**Baseline age (yrs)**	**Follow-up period**	**Effect of time and group × time**	**Brain-behavior correlations**
Lichenstein et al. (2022)	20	2 yrs	**FA (group-by-time)** *lower ↑↑* cingulum in moderately extended cannabis vs control	n.s. onset age, duration and frequency
Becker et al. (2015)	20	2 yrs	**FA (group-by-time)** *lower* ↑↑ in SLF (next to junction with corticospinal tract), SLF (ext. to corpus callosum forceps *major*), superior frontal gyrus, in cannabis vs. controls. *trend lower* ↑↑ in corticospinal tract (adj. to precentral and postcentral gyri) *and* anterior thalamic radiations; superior fronto-occipital fasciculus (adj. to frontal operculum) *greater* ↑↑ in corpus callosum (ant.), thalamus (adj. to post.), in cannabis vs. controls. **RD (group-by-time)** *lower* ↑↑ in SLF, corticospinal tract and cingulum (post.), in cannabis vs. controls. *higher* ↑↑ corticospinal tract in cannabis vs. controls.	neg. cor. cannabis hits/past year and FA change of corticospinal tract, SLF/corpus callosum and forceps *major*. neg cor. max. frequency of cannabis hits/past year and FA change of SLF/corticospinal tract n.s. cor. age of onset and RAVLT
Epstein and Kumra (2015)	17	1.5 yrs	**FA (group-by-time)** ↓↓ inferior longitudinal fasciculus in cannabis and ↑↑ controls *trend* ↓↓ inferior fronto-occipital fasciculus in cannabis vs controls **FA (time)** ↓↓ inferior longitudinal fasciculus in cannabis users	Neg. cor. total days cannabis use days over time and ↓↓FA of inferior longitudinal fasciculus
Jacobus et al. (2013a)	18	3 yrs	**FA (time)** ↓↓ corpus callosum (splenium), inferior fronto-occipital fasciculus, anterior thalamic radiations, uncinate fasciculus, SLF, corona radiata (ant., sup.), internal capsule (posterior limb). ↓↓ corpus callosum (genu), anterior thalamic radiations, and SLF.	Pos. cor. change in global neurocognition over time and FA SLF at follow-up
Jacobus et al. (2013b)	18	1.5 yrs	-	Baseline ↓ FA in fornix and sup. corona radiata predicted more cannabis use days and delinquent/aggressive risk taking at follow-up.

Adj, adjacent; Ant, anterior; Cor, correlated; FA, fractional anisotropy; Mage, mean age; Max., maximum; neg, negative; n.s., non-significant Pos, positive; Post, posterior; RAVLT, Rey Auditory Verbal Learning Test; SLF, superior longitudinal fasciculus; Sup, superior; yrs, years; -, not reported, ↑↑ increase over time; ↓↓ decrease over time.

Other relevant information from each study can be found in Supplementary material. This data comprised: originally reported substance use metrics (Supplementary Figures 1–3), ethnicity and/or race in cannabis users and control groups (Supplementary Table 1); inclusion and exclusion criteria for cannabis use levels in cannabis users and control groups as some studies allowed for specific amounts/past cannabis use in their control groups (Supplementary Table 2); methods used to acquire diffusion-MRI images (Supplementary Table 3), and which variables were matched between groups or controlled for in the analyses (Supplementary Figure 4). All data were summarized by counting the number of studies endorsing specific features, and/or ranges of values and means, where relevant.

### 2.5. Risk of bias assessment

ER and AG assessed the risk of bias of the reviewed literature, *via* the National Institute of Health, National Heart, Lung, and Blood Institute—Quality Assessment Tool for Observational Cohort and Cross-Sectional Studies Tool (http://www.nhlbi.nih.gov/health-topics/study-quality-assessment-tools) (Supplementary Table 4). This tool evaluates the risk of bias (i.e., present, or absent) against 14 criteria. Results from the risk of bias assessment are outlined in Section 7 of the Supplementary material.

### 2.6. Additional data handling

A total of 14 manuscripts required additional handling and variations to the data extraction protocol. One manuscript (Bava et al., 2010) reported DTI comparisons that had been already presented in a previous paper (Bava et al., 2009). However, as this paper also presented the neurocognitive correlates associated with the white matter microstructural differences reported previously (Bava et al., 2010), additional correlational findings from the more recent study are also reported. As these manuscripts report on the same sample, details of these studies are reported together as necessary.

Two studies utilized data from the human connectome project (HCP) dataset (Orr et al., 2016; Manza et al., 2020), although these studies used different subsets of participants, therefore mitigating any similarities between results and the results from both studies have been reported.

One paper reported that a portion of a larger sample (Jakabek et al., 2016) had been utilized by another previously published paper also included in this review (Zalesky et al., 2012). However, the results of both papers were reported, given that additional participants were utilized in the later paper. Additionally, 4 papers utilized different subsamples from a larger longitudinal study (Bava et al., 2009; Jacobus et al., 2009, 2013a,b). These papers reported on subsets of participants from the same dataset and were also authored by the same or similar groups of authors. Four other papers included in this review were also published by overlapping groups of authors and samples (Yücel et al., 2010; Yucel et al., 2016; Gruber et al., 2011, 2014). Given this similarity in samples used across these research groups, there is a potential for the generalizability of the results to be compromised.

Two studies also incorporated samples with combined binge drinking and cannabis use (Jacobus et al., 2009, 2013a), and 1 other reported on concurrent heavy alcohol and cannabis use (Jacobus et al., 2013b). However, given the high incidence of binge drinking in adolescents and young people, these studies were retained in the review. Finally, 2 papers reported on the same longitudinal cohort of participants, but they separately reported group differences at baseline (Epstein et al., 2014), and then at 18 months follow-up, and also the changes in white matter in both groups over time (Epstein and Kumra, 2015).

## 3. Results

A total of 2,712 studies were retrieved, and after duplicates (*n* = 1,429) were removed, 1,283 studies remained (see Figure 1). After screening, we included a total of 30 studies. In total 29 of 30 studies reported on white matter microstructural differences (Gruber and Yurgelun-Todd, 2005; Delisi et al., 2006; Arnone et al., 2008; Ashtari et al., 2009; Jacobus et al., 2009, 2013a,b; Bava et al., 2010; Yücel et al., 2010; Yucel et al., 2016; Gruber et al., 2011, 2014; Kim et al., 2011; Zalesky et al., 2012; Epstein et al., 2014; Filbey et al., 2014; Becker et al., 2015; Epstein and Kumra, 2015; Shollenbarger et al., 2015; Jakabek et al., 2016; Orr et al., 2016; Rigucci et al., 2016; Levar et al., 2018; Manza et al., 2020; Sweigert et al., 2020; Koenis et al., 2021; Cousijn et al., 2022; Knodt et al., 2022; Lichenstein et al., 2022). One of 30 studies reported brain-behavior correlations only (Bava et al., 2010). All studies were published between 2005 and 2022, and approximately 53% were published from 2014 onwards.

### 3.1. Overview of samples size and sex composition

Ultimately, the sample consisted of 2,898 participants. Of the studies that reported a sex distribution, this included 1,234 females (43.25%) and 1,620 males (56.75%). Within the total sample, 1,457 persons (562 females and 873 males) were cannabis users with a mean age of 24.2 years (range: 16.6 to 45.0 years) and 1,441 were controls (672 females and 747 males) with a mean age of 24.0 years (range: 16.6 to 45.0 years). Female participants were included in all but four studies that recruited male-only samples (Arnone et al., 2008; Ashtari et al., 2009; Kim et al., 2011; Lichenstein et al., 2022).

### 3.2. Overview of cannabis use levels

Data pertaining to cannabis use levels are presented (means and SDs) in Table 1. Importantly, participants began using cannabis, reported as either age of onset or age of first use, on average at age 15.4 years (range: 13.1 to 18.1 years). Nine studies reported age of ‘regular’ use, which was found to be on average 16.1 years (range: 14.5 to 17.9 years). The average duration of cannabis use amongst participants was 8.2 years (range: 3.4 to 15.7 years).

Across the sample, the average dosage of cannabis was 343.7 cones per month, corresponding to about 115 joints/month or approximately 4 daily joints (range: 76.5 to 763.9 cones per month). This was consumed on an average of 17.2 days per month (range: 3.4 to 25.7 days) or an average of 39.0 occasions per month (range: 11.2 to 81.5 occasions). Total lifetime occasions of cannabis use were reported for 2 studies; 1 study reported 471 occasions (Jacobus et al., 2013b), and lastly, 1 study as a categorical variable ranging from 1 to 5 occasions to 100+ occasions (Orr et al., 2016).

### 3.3. Overview of dMRI measures of white matter microstructure

All studies described in this review primarily used 4 different diffusion-MRI metrics of white-matter microstructure, which are described in Table 2. The diffusion-MRI metrics included: FA (27 studies), MD (also known interchangeably as ADC/Trace, 16 studies), RD (11 studies), and AD (10 studies). Novel diffusion-MRI techniques for investigating white matter microstructure were also utilized, and they are also defined in Table 2. In addition, methods of diffusion-MRI analyses that are utilized in the wider literature are also defined in the table.

These diffusion-MRI metrics are derived from the tensor model of the diffusion-MRI and vary in a way that provides specific interpretations of the white matter (Pierpaoli and Basser, 1996; Alexander et al., 2011). FA is highly sensitive to microstructural changes and provides a summary measure of white matter characterization at the microstructural level but does not assign the changes to specific features of the tissue microstructure without further assumptions. Alternatively, MD provides an inverse measure of membrane density and fluid viscosity providing a more biological measure of white matter characterization. Finally, RD and AD provide direct measures of more macrostructural elements of the white matter including axonal density (i.e., RD) and overall axonal caliber not influenced by myelin (i.e., AD). These diffusion-MRI metrics are often complementary to one another and taken together, they can provide an overall metric of the integrity of the brain's white matter.

### 3.4. Results pertaining to white matter differences between cannabis users and controls

**Figure 2:**
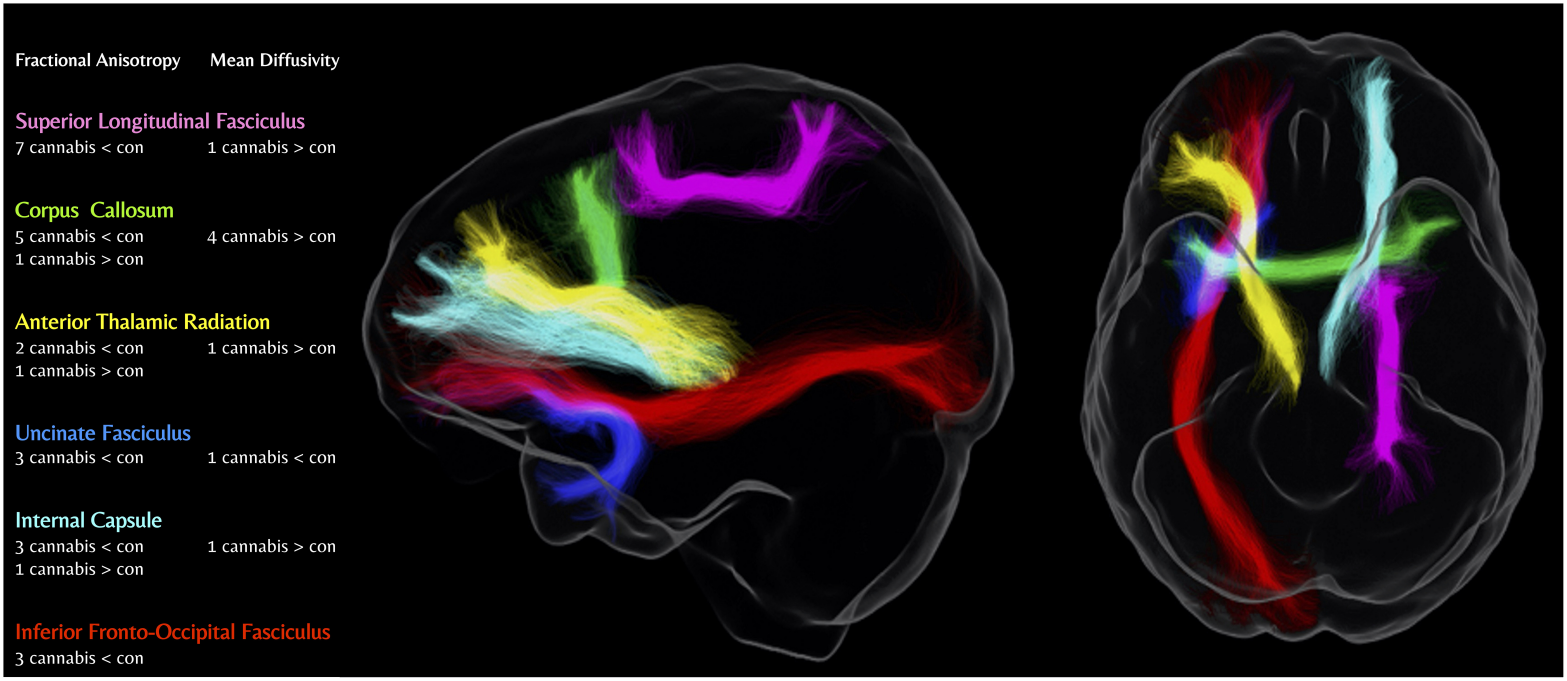
Number of diffusion-weighted MRI studies showing differences in major white matter tracts in cannabis users compared to controls. Cannabis < Con; Lower metric of white matter microstructure in cannabis users vs. controls, Cannabis > Con; Higher metric of white matter microstructure in cannabis users vs. controls. Figure produced using MRtrix3 and tracts generated from TractSeg (Wasserthal et al., 2018; Tournier et al., 2019).

This section summarizes white-matter differences between cannabis users and controls, as a function of the diffusion-MRI metric used (i.e., FA, MD, RD, and AD). Within each section below, findings are synthesized by brain region and direction of the difference (e.g., lower, higher, and both). A visual summary of the most consistent results is given in Figure 2. Specifically, Figure 2 focuses on the most consistent findings in the literature, which are prevalent for the FA and MD metrics only. As seen in the figure, there are consistent findings of significantly lower FA in cannabis users compared to controls in the SLF and the corpus callosum, followed by the uncinate fasciculus, inferior fronto-occipital fasciculus, internal capsule, and anterior thalamic radiations. MD is consistently found to be significantly greater in cannabis users compared to controls in the corpus callosum.

In addition to the figure, the overall findings for FA, MD, RD, AD, and non-tract-specific metrics (e.g., global network metrics) are summarized in Table 3. As can be seen, results are primarily detected in FA and MD metrics. The following section outlines further detail in relation to the findings from Table 3 and outlines the group differences in (1) FA; (2) MD; (3) RD; (4) AD; and (5) global network metrics. Within each section, there is an outline of the most consistent white matter tracts implicated in cannabis use for each diffusion-MRI metric, followed by a summary of the overall findings (if applicable). Details for consistent results within the literature (i.e., tracts implicated in at least 4 studies for 1 diffusion-MRI metric) are presented below. For tracts implicated in 3 or fewer studies, see Section 5 of the Supplementary material for further descriptions.

### 3.5. Group differences in *Fractional Anisotropy*

As discussed previously, FA is highly sensitive to microstructural changes and provides a summary measure of white matter characterization at the microstructural level (Pierpaoli and Basser, 1996). Overall, 17 of 27 studies found group differences in FA in cannabis users compared to controls (Table 4). For 11 studies, cannabis users had significantly lower FA than controls. Interestingly, 6 studies found higher FA in cannabis users in partially overlapping areas. Of these, 1 study reported higher and lower FA in multiple fibre tracts (Bava et al., 2009). Overall, the consensus of these findings is that cannabis users have lower FA compared to controls, except for in a few studies.

Multiple tracts showed significant group differences in FA. The most consistent findings were within the arcuate/SLF, corpus callosum, and internal capsule, which were shown to be significantly different in cannabis users in at least 4 studies. In addition, there were multiple tracts implicated in 3 or fewer studies that were not as consistent in the literature.

This section will further disentangle which white matter tracts were consistently found to have differences in FA (i.e., in 4 or more studies). This includes the (1) Arcuate/SLF; (2) corpus callosum; and (3) internal capsule. For tracts implicated in 3 or fewer studies, see Section 5 of Supplementary material; however, it is important to note, that these findings are inconsistent within the literature.

#### 3.5.1. Superior longitudinal fasciculus/arcuate

Seven studies examined the SLF or arcuate fasciculus (part of the SLF) and found significantly lower FA in cannabis users compared to controls (Ashtari et al., 2009; Bava et al., 2009; Jacobus et al., 2009, 2013b; Yucel et al., 2016; Manza et al., 2020; Koenis et al., 2021). One study found higher FA in the arcuate portion of the SLF (Bava et al., 2009).

#### 3.5.2. Corpus callosum

Five studies reported lower FA in the corpus callosum in cannabis users compared to controls (Bava et al., 2009; Gruber et al., 2011, 2014; Jacobus et al., 2013b; Manza et al., 2020). In addition, Becker et al. (2015) reported higher FA in the corpus callosum in cannabis users compared to controls only at baseline.

#### 3.5.3. Internal capsule

Four studies detected group differences in FA of the internal capsule. Of these, 3 studies found lower FA in cannabis users compared to controls (Ashtari et al., 2009; Jacobus et al., 2013a; Gruber et al., 2014). In contrast, 1 study detected higher FA in controls compared to cannabis users (Bava et al., 2009). Overall, the internal capsule was implicated consistently in studies examining differences in FA, though the direction of the differences varied between studies.

### 3.6. Group differences in *Mean Diffusivity*

As outlined in Table 1, MD provides an inverse measure of membrane density and fluid viscosity providing a measure of white matter characterization that is complementary to FA [which does not provide biological specificity (Pierpaoli and Basser, 1996)]. Sixteen studies compared MD between cannabis users and controls, and of these 10 studies found differences (Table 5). The most consistent finding was higher MD in cannabis users compared to controls in 7 studies. In contrast, 2 studies reported lower MD in cannabis users compared to controls (Delisi et al., 2006; Sweigert et al., 2020). Interestingly, 1 of these studies also found lower MD across different white matter tracts, in addition to the higher MD found (Bava et al., 2009).

Of the 7 studies that found higher MD in cannabis users, a total of four studies found higher MD in the corpus callosum (Arnone et al., 2008; Gruber et al., 2011, 2014; Rigucci et al., 2016). There were multiple studies that found individual tract differences in MD, see Section 5 of the Supplementary material for a discussion of these tracts.

Overall, the literature shows a consistent finding of higher MD in cannabis users compared to controls. Although there was heterogeneity in the findings linked mostly to single tracts, 57% of studies implicated the corpus callosum with poorer white matter integrity in cannabis users.

### 3.7. Group differences in *Radial Diffusivity*

As described earlier, RD provides a metric related to the integrity of white matter macrostructure (Alexander et al., 2011). There were no consistent findings in RD across all studies when measuring white matter integrity of cannabis users compared to controls. Less than half of the studies that examined RD found group differences (i.e., 5 out of 11, Table 6). The direction of the differences was mixed, 2 studies found higher RD in various tracts (e.g., arcuate fasciculus, internal capsule/thalamic radiation) (Ashtari et al., 2009; Rigucci et al., 2016) and another 3 studies showed lower RD in cannabis users compared to controls in the corpus callosum (Becker et al., 2015), forceps minor (Filbey et al., 2014), and middle cerebellar peduncle (Sweigert et al., 2020). Overall, this metric (1) was not as frequently used in the literature compared to other diffusion-MRI metrics, such as FA and MD and (2) showed large heterogeneity across findings in the white matter tracts and directionality of the results, with no clear outline on higher or lower white matter integrity of cannabis users when compared to controls.

### 3.8. Group differences in *Axial Diffusivity*

AD also provides a measure of white matter macro-structural integrity, as presented in Table 1 (Alexander et al., 2011). There were no consistent findings in AD across the studies when measuring white matter changes in cannabis users compared to controls. Only 2 of 10 studies that examined AD, found group differences (Table 7). One study found cannabis users had higher AD in the temporal lobe, internal capsule/thalamic radiation, and motor tracts (Ashtari et al., 2009), and 1 study found cannabis users had lower AD in the corpus callosum (Rigucci et al., 2016). Overall, these findings indicate that AD: (1) is not as frequently used compared to other diffusion-MRI metrics such as FA and MD; (2) did not detect differences in the majority of studies; and (3) there is heterogeneity in the findings across the 2 studies and differences in the directionality of the findings, with no clear consistency in the findings.

### 3.9. Group differences in other DTI metrics of white matter integrity

It is important to note that not all studies measured tract-specific quantifications of white matter integrity. A minority of studies focused on other metrics including (1) global network metrics of the white matter pathways; (2) metrics on the number of streamlines in the white matter bundles; and (3) fibre bundle length. The descriptions of these single studies can be found in Section 5 of the Supplementary material. Overall, it is important to note that these metrics are not widely used in the cannabis use literature, and there are no consistent findings reported.

### 3.10. Overview of *correlations* between diffusion-MRI metrics and indices of cannabis use, cognitive, alcohol use, and mental health-related variables

Table 8 shows that 15 of 30 studies investigated correlations between diffusion-MRI metrics (i.e., FA, MD, RD, AD, and other non-tract specific metrics) and indices of cannabis use (e.g., age of onset, duration, dosage, frequency, cannabis dependence severity, and abstinence), as well as other key cognitive, alcohol use, and mental health-related variables.

Overall, 2 indices of cannabis use were consistently measured to determine their associations with white matter integrity. This includes (1) age of onset; and (2) duration of use. There were two main implications of these findings. First, although FA, MD, RD, and AD were measured frequently to determine their associations with age of onset and duration of use, ~50% of the studies did not detect any significant associations. Secondly, most of the significant findings indicated that reduced white matter microstructure across all measures was associated with (1) lower age of onset and (2) longer duration of use. These important findings are comparatively rare in the literature as not all potential correlations are investigated.

The findings which were not consistently reported within the studies reviewed (i.e., in 3 or fewer studies per metric) are described in Section 6 of the Supplementary material. These findings have several implications pertaining to (1) the lack of consistency in running correlations for these outcomes and diffusion-MRI metrics; (2) the lack of associations detected; and (3) the need to further include these analyses in future studies to ensure robust comparisons can be determined.

#### 3.10.1. Metrics for cannabis exposure

The following sections summarize correlational findings between cannabis exposure metrics and diffusion-MRI metrics (FA, MD, RD, and AD) when there are more than 3 studies for each metric.

##### 3.10.1.1. Age of cannabis use onset and *Fractional Anisotropy*

Nine studies investigated the correlation between age of onset of cannabis use and differences in FA, with four of these studies finding significant positive correlations. There were consistent findings across the literature in the corpus callosum and the ILF across 4 studies with significant findings, as shown in Table 8. In other words, earlier age of onset of cannabis use was positively correlated with lower FA in the corpus callosum (genu) in 3 studies (Gruber et al., 2011, 2014; Orr et al., 2016). In addition, the 2 studies found positive correlations between age of onset and FA in the ILF (Orr et al., 2016; Cousijn et al., 2022). Together, these findings indicate the earlier onset of cannabis use is associated with reduced FA in corpus callosum and ILF among cannabis users.

##### 3.10.1.2. Age of cannabis use onset and *Mean Diffusivity*

Five studies investigated the correlation between age of onset of cannabis use and differences in MD, with only one study showing significant findings. Gruber et al. (2011) found that age of onset is negatively correlated with MD in the genu and white matter tracts within the left frontal region. This finding indicates that earlier onset of use is associated with reduced white matter integrity, however, this is not consistently found across the literature.

##### 3.10.1.3. Age of cannabis use onset and *Radial Diffusivity*

Four studies investigated correlations between age of onset of cannabis use and RD, with 2 studies showing significant findings in various regions. Age of onset was positively correlated with RD in the commissural fibre (beginning of the splenium and extending with the pre-cuneus) and fimbria (Zalesky et al., 2012) and was negatively correlated with RD in the SLF, lateral prefrontal white matter, corpus callosum (anterior and posterior), and ILF (Orr et al., 2016). These findings indicate heterogeneity in the findings with early onset of use associated with both higher and lower white matter integrity as measured by RD.

##### 3.10.1.4. Age of cannabis use onset and *Axial Diffusivity*

A total of five studies investigated the correlations between age of onset of cannabis use and AD, with two studies having significant findings in several white matter tracts. Age of onset of cannabis use was significantly positively correlated with AD in the commissural fibre and with AD in the fimbria, albeit at a trend level (Zalesky et al., 2012). Age of onset was also negatively correlated with AD in the cingulate gyrus (Jakabek et al., 2016). Similar to RD, there was a large heterogeneity in findings in AD, with early age of onset being associated with higher and lower white matter integrity.

##### 3.10.1.5. Duration of cannabis use and *Fractional Anisotropy*

Six studies investigated the correlations between the duration of cannabis use and FA, with 3 of these showing significant findings. In 2 studies FA was negatively correlated with duration of cannabis use in the corpus callosum (genu) (Gruber et al., 2011), and the ILF (Jakabek et al., 2016). That is, poorer white matter integrity was associated with longer duration of cannabis use. Additionally, there was a significant non-linear quadratic relationship between FA in the forceps minor and duration of cannabis use (i.e., gains with initial heavy use, but declined after chronic use (Filbey et al., 2014). Overall, these findings indicate a consistent relationship between longer cannabis use duration and the corpus callosum, which were heterogeneous by specific sub-sections of the corpus callosum across the studies.

##### 3.10.1.6. Duration of cannabis use and *Mean Diffusivity*

Four studies investigated the correlations between the duration of cannabis use and MD, with two studies having significant findings. Both studies found there was a positive correlation between duration of cannabis use and MD in the corpus callosum (at a trend level) (Arnone et al., 2008) as well as the genu of the corpus callosum (Gruber et al., 2011). Similar to FA, this indicates a consistent finding of reduced white matter integrity being associated with longer cannabis use duration—particularly across sub-sections of the corpus callosum.

##### 3.10.1.7. Duration of cannabis use and *Radial and Axial Diffusivity*

Five studies investigated the correlations between the duration of cannabis use and RD, with 2 of these showing significant findings. These included a negative correlation between the duration of cannabis use and RD in the cingulum angular bundle (Jakabek et al., 2016), as well as a quadratic relationship between RD in the forceps minor, and duration of use (i.e., gains with initial heavy use, but declined after chronic use) (Filbey et al., 2014). These findings indicate that decreased white matter integrity is consistently associated with less duration of cannabis use. Moreover, 2 studies investigated the correlations between the duration of cannabis use and AD, however, non-significant findings were observed.

#### 3.10.2. Other non-consistent findings on white matter associations with cannabis use indices

Some studies showed correlations between diffusion-MRI metrics and indices of cannabis use that were not consistently found in the literature (i.e., found in 3 or fewer studies). Descriptions of these studies can be found in Section 6 of the Supplementary material. To summarize, these studies assessed correlations between (1) age of onset and other metrics (e.g., graph theory measures) and (2) dosage, frequency, abstinence, and dependence severity and its associations with all diffusion-MRI metrics of white matter integrity (e.g., Zalesky et al., 2012).

#### 3.10.3. Cognition, alcohol use, and mental health

Overall, there were no consistent correlations between any diffusion-MRI metric and any measures of cognition, alcohol use, and mental health outcomes (i.e., 4 or more studies). However, some studies found associations between these variables, in up to 3 studies, which are described in Section 6 of the Supplementary material. For cognition, alcohol use, and mental health-related variables, these included studies on impulsivity, neurocognitive performance, memory, alcohol use, and mental health (i.e., depression and anxiety) and all measures of white matter integrity.

### 3.11. Overview of the effects of time and group-by-time from longitudinal studies

Five of the 30 studies included were longitudinal studies that examined white matter integrity, with details outlined in Table 9. Three studies reported significant group-by-time effects in several white matter pathways (Becker et al., 2015; Epstein and Kumra, 2015; Lichenstein et al., 2022), and additional interesting findings emerged. Overall, there was no consistency across these significant findings across the literature in terms of direction of findings and white matter tracts implicated, longitudinally. Two of these studies reported a lower increase in white matter integrity over time in cannabis users compared to healthy controls (Becker et al., 2015; Lichenstein et al., 2022), however these were in different white matter tracts [i.e., SLF, cingulum, and Corticospinal tract (CST) (trend)]. Interestingly, FA was the most sensitive metric to longitudinal changes in group-by-time effects, being implicated in all three studies, with RD only being implicated in one study (i.e., Becker et al., 2015).

For the effects of time, only two studies found significant differences, which were both decreases in white matter integrity in cannabis users (Jacobus et al., 2013a; Becker et al., 2015). These differences were only found in the FA metric and were implicated in a wide array of tracts, not consistent between both studies.

Finally, there were no consistent findings of brain-behavior correlations between measures of change in white matter integrity and cannabis-related metrics. Two studies showed negative correlations between cannabis-related metrics (e.g., cannabis hits/past year, total days cannabis use over time) and white matter integrity (Becker et al., 2015; Epstein and Kumra, 2015). However, the white matter tracts implicated were not consistent across the studies.

## 4. Discussion

Overall, the diffusion-MRI literature to date largely shows significant white matter microstructural differences between cannabis users and controls (all but 6 of the 30 studies reviewed here). The most consistent diffusion-MRI metric reported to be different between cannabis users and controls was Fractional Anisotropy (FA)—which was lower in cannabis users than controls (i.e., poorer integrity) in 12 studies—followed by 8 studies for Mean Diffusivity (MD), 3 studies for Radial Diffusivity (RD), and 2 studies for Axial Diffusivity (AD). These structural differences were seen most consistently in the Superior Longitudinal Fasciculus (SLF)/Arcuate Fasciculus, with largely lower FA seen in 7 studies, followed by the corpus callosum (with 5 studies showing decreased FA, 1 study showing increased FA, and 4 studies showing increased MD).

Additionally, multiple studies investigated correlations between white matter integrity and cannabis exposure metrics. Most commonly, 4 out of 9 studies found positive correlations between FA and age of onset [especially in the corpus callosum (3 studies) and ILF (2 studies)]. In addition, there were correlations detected between duration of cannabis use, and FA in the corpus callosum, ILF, and the forceps *minor*. Preliminary changes were also seen in cannabis users longitudinally in 2 of 3 studies, including in the SLF/CST (correlating with maximum cannabis use frequency), and SLF/corpus callosum forceps *major* junction and CST (correlating with total cannabis hits over time). Finally, decreased FA was also seen in the ILF over time (correlating with days of cannabis exposure between baseline and follow-up).

### 4.1. Superior longitudinal fasciculus

The SLF was one of the most consistent pathways with white matter differences between groups. The SLF, along with the arcuate fasciculus, is a major association pathway in the brain, connecting the frontal lobes with the ipsilateral parietal, occipital, and temporal lobes (Schmahmann et al., 2008). It has been implicated in executive functioning, including sustained attention (Clemente et al., 2021), and the frontal mediation of attention and executive function (Baker et al., 2013). Importantly, 2 studies found correlations between differences in the SLF, and cannabis exposure metrics, a positive association between FA and age of onset, and a negative association between RD and age of onset, in addition to a positive correlation between FA and frequency of use (hits/past 3 months). Furthermore, in a study of children and adolescents, FA in the SLF was positively correlated with cognitive set-shifting, an important domain of executive functioning (Urger et al., 2015). As such, it could be suggested that decreased FA within the SLF of cannabis users may also be associated with decreased executive function, especially considering that early onset (Gruber et al., 2012), and exposure to high-potency cannabis (Ramaekers et al., 2006) has been associated with decreased executive function. Based on the evidence above, we may infer a potential cumulative relationship between cannabis use and differences in SLF microstructure. However, given how few studies indicated significant associations between SLF microstructure, and cannabis exposure metrics, it is difficult to ascertain if these differences are neuroadaptations associated with the effects of cannabis on the brain or, are instead differences in integrity, perhaps related to executive function, that predate cannabis use onset. Longitudinal diffusion-MRI research with careful assessment of cannabis exposure metrics is warranted to elucidate the role of cannabis exposure on SLF microstructure.

### 4.2. Corpus callosum

Differences in white matter microstructure were also consistently seen in the corpus callosum, with differences in both FA and MD. The corpus callosum is a major commissural tract that allows for inter-hemispheric communication (Standring and Gray, 2021). Across multiple studies, correlations were shown between white matter differences in the corpus callosum and cannabis exposure metrics. Associations were seen between microstructure of the corpus callosum and age of onset (Gruber et al., 2011, 2014; Orr et al., 2016), duration of use (Arnone et al., 2008; Gruber et al., 2011), and frequency of use (Rigucci et al., 2016).

In a previous meta-analysis of white matter microstructure, differences in FA in the corpus callosum have been noted between persons who use substances (e.g., cannabis, alcohol, nicotine, and opiates) and controls (Hampton et al., 2019). Such group differences in white matter microstructure might reflect neuroadaptations from addiction processes shared across different substances (Hampton et al., 2019). Additionally, a recent study of medicinal cannabis use, found increased FA in the corpus callosum after 3 and 6 months of administration (Dahlgren et al., 2022). Although medicinal and recreational cannabis use differ, this finding nonetheless suggests that cannabis use may alter corpus callosum microstructure.

Studies into CB1 receptor density in rats have shown that high receptor density in white matter tracts, including the corpus callosum, is present early in development, before decreasing and becoming denser in gray matter areas into adulthood (Romero et al., 1997). Adolescence is a period characterized by large-scale neurodevelopment and major brain maturational changes (Asato et al., 2010). It is also, concurrently, a time during which cannabis use may first commence (Richmond-Rakerd et al., 2017; AIHW, 2020). Further, the endocannabinoid system—which comprises CB1 and CB2 receptors, multiple endogenous lipid derivatives which activate them, as well as enzymes which control the levels of the lipid derivatives—plays a key role in typical neurodevelopment (Malone et al., 2010; Hourani and Alexander, 2018). Therefore, white matter differences observed in cannabis users may reflect the influence of exogenous cannabis exposure on the endocannabinoid system that play a key role in neurodevelopment.

The exact mechanisms by which cannabis use may be associated with differences in white matter integrity are largely unclear. Correlations between white matter and cannabis dosage suggest neuroadaptations from exposure to cannabis as postulated by proponents of neuroscientific theories of addiction (Koob and Volkow, 2016; Zehra et al., 2018); possible neurotoxic effects preliminarily shown in animal studies (Scallet, 1991; Sarne et al., 2011); while correlations between the age of cannabis use onset and FA may reflect developmental processes affected by cannabis exposure through brain maturation, possibly *via* cannabinoids affecting the endocannabinoid system that regulates neurodevelopmental processes (Meyer et al., 2018; Farrelly and Vlachou, 2021). Yet, the exact mechanisms that underlie such associations are yet to be clarified with human and preclinical studies. While there is some evidence to suggest that corpus callosum microstructural differences are associated with cannabis exposure, longitudinal studies that capture brain maturation prior to the onset of cannabis use, are necessary to understand which parameters drive white matter changes in cannabis users.

### 4.3. Non-significant group differences

A minority of 5 studies did not find significant cross-sectional group differences in DTI metrics (Jacobus et al., 2013b; Orr et al., 2016; Yucel et al., 2016; Knodt et al., 2022; Cousijn et al., 2022). One study reported only trend level differences in white matter microstructure (Gruber and Yurgelun-Todd, 2005). In addition, another study found non-significant differences in diffusion-MRI metrics, finding significant differences in fibre bundle length, but not the DTI metrics FA, MD, RD, or AD (Levar et al., 2018). The lack of group difference may be due to moderate levels of cannabis exposure in the control groups [e.g., up to 50 cannabis use occasions (Cousijn et al., 2022)], low levels of cannabis exposure in the cannabis samples [e.g., < 10 times in 50% of the sample (Orr et al., 2016), or recreational levels of use (Levar et al., 2018)]. It also cannot be excluded that the examined samples endorsed additional features that are protective of cannabis-related neuroanatomical changes, such as youth age (Solowij et al., 2016a; Lorenzetti et al., 2021), unmeasured lifestyle and physiological indices, known to affect neuroanatomy [e.g., exercise and body composition (Kandola et al., 2016; Den Ouden et al., 2018; Kakoschke et al., 2019)].

### 4.4. Limitations of reviewed studies

In understanding these heterogeneous findings, it is important to note that many of the reviewed studies were subject to several limitations. This section will detail the limitations of these studies, which include and are not limited to small sample sizes; presence of cannabis use in control groups and low cannabis use in cannabis groups; heterogeneity of measures and analyses; limited exploration of key variables correlating with white matter microstructure and the intrinsic limits associated with diffusion-MRI metrics due to the lack of biological specificity in DTI metrics.

#### 4.4.1. Small sample sizes

Issues with sample size were evident across multiple studies (Table 1). A pilot study of cannabis users incorporated 9 heavy cannabis users and 9 controls (Gruber and Yurgelun-Todd, 2005). This study detected only trend-level differences in trace in the corpus callosum, and the anterior cingulate gyrus. Another paper with a sample size of only 11, whilst showing significant differences in MD, also failed to show the differences in FA in the corpus callosum (Arnone et al., 2008) as evidenced in multiple other recent larger papers (Bava et al., 2009; Gruber et al., 2011, 2014; Jacobus et al., 2013b; Becker et al., 2015). These smaller sample sizes may account for the lack of statistical differences in cannabis users vs. controls, especially in relation to the detection of small effect sizes.

#### 4.4.2. Accounting for cannabis use in control groups

The reviewed studies used inconsistent inclusion and exclusion criteria pertaining to cannabis use levels in cannabis users and control groups. Out of the 30 studies, 6 studies clearly excluded lifetime cannabis use in their control groups, 2 studies did not clearly specify other than “non-users/healthy controls”, or not “regular users”, and 2 more did not outline cannabis use requirements. Fifteen studies allowed for specific amounts/past cannabis use in their control groups (Supplementary Table 2). Allowing cannabis use in control groups may be important in producing representative research in specific populations. For example, having used cannabis in controls (in limited amounts) might be important to prevent that controls and cannabis groups do not have systematic differences in variables entrenched with access to cannabis (Cousijn et al., 2022). Meanwhile, the effects of low-level cannabis use at a young age need to be accounted for. We warrant future studies to carefully measure cannabis use in all groups and to account for it as required to address the target research questions.

#### 4.4.3. Heterogeneity of cannabis exposure metrics

The heterogeneous cannabis exposure metrics preclude systematic integration of the findings to elucidate the role of cannabis exposure on white matter integrity in cannabis users. All measures of cannabis use were converted to a standardized unit of measurement of “cones” (where possible), to allow for easier comparison within the review. However, this is only an approximate representation of cannabis use in each sample, especially when the amount of cannabis in a joint, a commonly reported measure, may be significantly different between and within users. Additionally, several studies used subjective measures of cannabis use that are difficult to standardize, for example, “occasions” or “episodes” of substance use, which may provide poor precision in relation to quantity making comparisons between studies difficult. We recommend that future research utilizes standardized cannabis exposure metrics to enable the systematic integration of findings and their interpretation (Lorenzetti et al., 2021).

#### 4.4.4. Lack of assessment of how cannabis exposure/misuse metrics affect white matter microstructure

Emerging evidence does suggest an important relationship between the age of onset of cannabis use and differences observed in white matter microstructure, most notably FA, and most consistently in the corpus callosum. However, just over half of the studies (*n* = 15) investigated correlations between diffusion-MRI metrics and cannabis exposure metrics. For example, of the 27 papers using FA as a metric, and of the 22 studies that also reported age of onset, only 9 studies investigated correlations between FA and age of onset, with 4 revealing significant positive correlations. As such, there is the potential for non-significant results to be under-reported, and findings relating cannabis exposure metrics to white matter microstructure should be interpreted carefully. Pre-registration of analyses in future studies might be instrumental in ensuring the reporting of non-significant findings. Further, cannabis dependence and cannabis use disorder have been poorly measured and assessed in relation to the diffusion-MRI data, with only 3 studies utilizing standardized diagnostic criteria (Ashtari et al., 2009; Manza et al., 2020; Koenis et al., 2021). Given that cannabis use-related problems have been shown to affect brain integrity, future diffusion-MRI studies are required to explore if they moderate white matter integrity changes as shown for other measures of brain integrity (Lorenzetti et al., 2016b, 2020a; Manza et al., 2020; Rossetti et al., 2021a).

#### 4.4.5. Heterogeneity of study analyses

An additional limitation was the lack of consistency between studies in which potentially confounding variables were controlled for in analyses, as outlined in the Supplementary Table 4. Studies routinely reported potentially confounding variables, including age, sex, handedness, intelligence (often IQ), alcohol use, tobacco use, and other substance use. However, studies did not consistently control for these in analyses, or match participants on these variables (i.e., studies may have controlled for/matched many, one, or none of these and other variables). These are variables that are themselves frequently associated with differences in white matter microstructure, such as age and sex (Grace et al., 2021; López-Vicente et al., 2021; Rossetti et al., 2021b), alcohol (Rossetti et al., 2022), nicotine (Gogliettino et al., 2016), IQ (Suprano et al., 2020), and other substance use (Hampton et al., 2019; Rossetti et al., 2022). In order to limit the confounding effects of these variables, it is important they are systematically controlled for or at least measured if not feasible to match, to allow for greater comparability of results.

#### 4.4.6. Limitations of longitudinal studies

Five studies carried out longitudinal analyses related to white matter microstructure in cannabis users. Importantly, none of these studies investigated white matter microstructure prior to the onset of cannabis use, and therefore do not provide insight into what differences in white matter microstructure existed prior to the use of cannabis (i.e., differences which may underlie risk factors for substance use behaviors). Therefore, longitudinal research is needed to measure white matter integrity both before and after cannabis use commences to delineate with precision the effects of cannabis on the brain over time.

#### 4.4.7. Limited assessment of diffusion-MRI metrics

DTI metrics (e.g., FA, MD, RD, and AD) are the most commonly examined white matter measures in the current studies. Despite this, these metrics are limited in that they rely on averaging values across a voxel, as the single tensor represented in DTI can only represent a single fibre direction per voxel (Tournier et al., 2007). This is problematic in areas with “crossing fibres”, that is, any area that has multiple fibres with different orientations within a single voxel (Farquharson et al., 2013). This is potentially problematic as to 90% of the white matter within the brain potentially being made up of such “crossing fibres” (Jeurissen et al., 2013). Whilst MD/ADC/trace are largely unaffected by this issue, voxel averaged measures, including FA, RD, and AD are particularly impacted, and can lead to errors in interpretation of white matter microstructure (Tournier et al., 2011). This includes counterintuitive results, such as an increase in white matter integrity when there may have been a decrease (Farquharson et al., 2013).

As such, additional studies may be needed using more sophisticated tools to delineate subtle alterations to white matter. One such technique is Constrained Spherical Deconvolution (CSD) based methods such as Fixel Based Analysis (FBA) (Tournier et al., 2019). CSD produces a fibre orientation distribution, without the need to assume the number of fibre populations present, which accounts for “crossing fibres” (Tournier et al., 2004). This provides a more biologically specific quantitative measure of white matter integrity which overcomes the major drawbacks of the DTI model. For the FBA framework—the fibre orientation distribution where a “fixel” represents the overall fibre population within a single voxel (Raffelt et al., 2015), can then be used to calculate microstructural fibre density, macrostructural fibre-bundle morphology (cross-section), and a combined measure of fibre density and cross-section.

A recent review of all studies using FBA identified no research using FBA in substance-using populations, however other populations (e.g., older people, children and people diagnosed with psychiatric disorders) have been investigated using this framework (Dhollander et al., 2021). Given the greater specificity of FBA in identifying tracts of interest, especially in areas with crossing fibres, it will become increasingly relevant to measure alterations to white matter microstructure with more recently developed techniques that overcome the drawbacks of DTI, such as FBA. This may account for some heterogeneity in the literature, especially with those minority findings found in opposite directions (e.g., greater white matter integrity in cannabis users compared to controls). Race or ethnicity or both may have played a role in brain changes in cannabis users. While we did not observe systematic differences between studies' results by race or ethnicity, it cannot be excluded that race or ethnicity or both affected brain changes in cannabis using participants, as their influence is yet to be systematically assessed.

Furthermore, there have been improvements in diffusion-MRI methodology triggered by the advent of high-performance MRI gradients technology after 2014 (Sotiropoulos et al., 2013). We were unable to identify any systematic differences in results between studies published prior to 2014, compared to those published during or after this time. In short, there was a consistent proportion of significant findings in relation to corresponding white matter tracts. The lack of systematic differences in findings pre-to-post technological advances in 2014 may be due to a number of factors, including that not all studies incorporated improvements in their diffusion parameters, and the high prevalence of DTI metrics within the literature.

### 4.5. Limitations of the review

This review includes several limitations that need to be considered. For example, the reviewed studies might have incorporated participants endorsing major mental health problems or significant exposure to substances other than cannabis. Indeed, we screened for studies endorsing these exclusion criteria *at a group level*. Thus, it cannot be excluded that the literature findings might have been partially affected by other variables that also affect brain integrity, including mental health problems or exposure to substances other than cannabis or both (Brambilla et al., 2003, 2005; Squarcina et al., 2017; Prunas et al., 2018; Mackey et al., 2019; Chye et al., 2020b; Delvecchio et al., 2020; Navarri et al., 2020; van Velzen et al., 2020; Cao et al., 2021; Rossetti et al., 2022; Zovetti et al., 2022). This issue might have biased the findings, or else might have rendered the findings more representative of persons who use cannabis. Indeed, some groups of cannabis users—such as those endorsing a cannabis use disorder—experience mental health problems and polysubstance use (Lawn et al., 2022).

Further, we included samples exposed to alcohol, in particular, 2 manuscripts included persons who used cannabis and engaged in binge drinking (Jacobus et al., 2009, 2013b). Importantly, alcohol exposure can affect white matter integrity independently and in interaction with cannabis (Rossetti et al., 2022). Indeed, binge drinkers with vs. without cannabis use showed differences in selected white matter tracts [e.g., uncinate fasciculus (Jacobus et al., 2013b)]. Given that cannabis and alcohol use often co-occur (AIHW, 2022), the findings may reflect neurobiological changes of exposure to both substances. Future work is warranted to measure alcohol exposure and account for it in analyses of white-matter integrity in cannabis users, to unpack the impact of co-occurring alcohol and cannabis exposure.

Finally, the review did not include samples who consumed synthetic cannabinoids, due to their different psychopharmacological profile of synthetic cannabinoids vs. cannabinoids (van Amsterdam et al., 2015). Synthetic cannabinoids can also affect white matter integrity (Zorlu et al., 2016; Gokharman et al., 2020) and are becoming increasingly diversified and available. Therefore, further research is required to examine how synthetic cannabinoids affect white matter microstructure.

### 4.6. Future directions and research

The reviewed evidence warrants recommendations for future work. First, the evidence reports heterogeneous metrics and findings (e.g., distinct measures of brain integrity and brain pathways). Future studies are warranted to apply precise and robust analytical frameworks in order to identify subtle changes to white matter in cannabis users. For example, FBA holds promise as a robust tool to map with precision white matter integrity as outlined above (Raffelt et al., 2017). Second, we warrant pre-registration of future empirical studies to encourage transparency in the reporting of the analyses and findings and reduce the risk of “cherry-picking” significant results and publication bias. Thus far, only 1 of the reviewed studies was pre-registered (Knodt et al., 2022).

Third, future studies should report cannabis use in a systematic and standardized fashion (Lorenzetti et al., 2016a,c; Solowij et al., 2016b; Lopez-Pelayo et al., 2021). They include standard THC units (Freeman and Lorenzetti, 2019, 2021) which have received support from researchers and health organizations internationally (Filbey, 2020; Hammond and Goodman, 2020; Volkow and Weiss, 2020; NIDA, 2021), as well as the iCannToolkit (Lorenzetti et al., 2021, 2022a,b), also endorsed by researchers internationally (Kuhns and Kroon, 2021; Weiss and Volkow, 2021). The use of standardized tools to measure cannabis exposure will enable the understanding of how specific parameters of cannabis exposure (e.g., age of onset, dosage, and duration) and levels of exposure might be risky for white matter integrity. Fourth, longitudinal multimodal neuroimaging studies research are necessary to determine when white matter changes begin to appear (e.g., before cannabis use onset, at specific times after cannabis use commences), how they change over time and if they dissipate after cessation of cannabis use. These research questions may be achieved *via* using data from large-scale longitudinal consortia initiatives (e.g., ABCD study).

## Conclusions

In the most up-to-date and pre-registered systematic literature review of diffusion-MRI studies thus far, we found that white matter differences between cannabis users and controls were consistently reported in select white matter tracts and diffusion-MRI metrics. The most consistently reported findings were lower FA of the SLF, and higher MD of the corpus callosum. Further, there was emerging evidence for an association between FA within the corpus callosum and the age of cannabis use onset.

However, longitudinal work is required to uncover how white matter integrity changes predate, follow and change as patterns of cannabis use vary over time, if white matter integrity changes in cannabis users exacerbate in those with more severe problems with use, the role of confounders entrenched with cannabis use (e.g., other substance use, elevated mental health symptoms). Ultimately, knowledge of which brain pathways show white matter microstructural alterations in cannabis users—and on the characteristics of those users who are most vulnerable to white matter changes is required to update prominent neuroscientific theories of addiction (Koob and Volkow, 2016).

## Data availability statement

The original contributions presented in the study are included in the article/Supplementary material, further inquiries can be directed to the corresponding author.

## Author contributions

ER conducted all the searches, screening, data selection, conceptualized the paper, created the first full draft, and edited it with co-authors' input. VL supervised all the aspects of the work including conceptualization and provided significant relevant edits on all aspects of the manuscript. HC supervised all the aspects of the work and provided significant comments. AC extracted diffusion-MRI data from studies, provided comments on the manuscript, created two tables, and provided significant comments. AG co-created risk of bias section, provided comments, and assisted with referencing. AA co-created one figure and provided comments. JG provided significant comments. MR, PB, CC, and MB provided comments. All authors contributed to the article and approved the submitted version.
