# Supplementary material for: Measuring white matter microstructure in 1,457 cannabis users and 1,441 controls: A systematic review of diffusion-weighted MRI studies
Source: Front Neuroimaging. 2023 Mar 7;2:1129587. doi: 10.3389/fnimg.2023.1129587 (PMC10406316; doi:10.3389/fnimg.2023.1129587)
Supplement: Supplementary file 1 [file Data_Sheet_1.docx]

Supplementary Material

**Measuring white matter microstructure in 1,457 cannabis users and 1,441 controls: A systematic review of diffusion-weighted MRI studies**

**Emily Anne Robinson, John Gleeson, Arush Honnedevasthana Arun, Adam Clemente, Alexandra Gaillard, Maria Gloria Rossetti, Paolo Brambilla, Marcella Bellani, Camilla Crisanti, H. Valerie Curran, Valentina Lorenzetti***

*** Correspondence:** A/Prof Valentina Lorenzetti, PhD: valentina.lorenzetti@gmail.com

# PRISMA checklist

| **Section and Topic** | **Item #** | **Checklist item** | **Location where item is reported** |
| --- | --- | --- | --- |
| **TITLE** | | |  |
| Title | 1 | Identify the report as a systematic review. | 1 |
| **ABSTRACT** | | |  |
| Abstract | 2 | See the PRISMA 2020 for Abstracts checklist. | 1-2 |
| **INTRODUCTION** | | |  |
| Rationale | 3 | Describe the rationale for the review in the context of existing knowledge. | 2 |
| Objectives | 4 | Provide an explicit statement of the objective(s) or question(s) the review addresses. | 2 |
| **METHODS** | | |  |
| Eligibility criteria | 5 | Specify the inclusion and exclusion criteria for the review and how studies were grouped for the syntheses. | 3&6 |
| Information sources | 6 | Specify all databases, registers, websites, organisations, reference lists and other sources searched or consulted to identify studies. Specify the date when each source was last searched or consulted. | 3 |
| Search strategy | 7 | Present the full search strategies for all databases, registers and websites, including any filters and limits used. | 3 |
| Selection process | 8 | Specify the methods used to decide whether a study met the inclusion criteria of the review, including how many reviewers screened each record and each report retrieved, whether they worked independently, and if applicable, details of automation tools used in the process. | 3 |
| Data collection process | 9 | Specify the methods used to collect data from reports, including how many reviewers collected data from each report, whether they worked independently, any processes for obtaining or confirming data from study investigators, and if applicable, details of automation tools used in the process. | 3&6-7 |
| Data items | 10a | List and define all outcomes for which data were sought. Specify whether all results that were compatible with each outcome domain in each study were sought (e.g., for all measures, time points, analyses), and if not, the methods used to decide which results to collect. | 3&6 |
|  | 10b | List and define all other variables for which data were sought (e.g., participant and intervention characteristics, funding sources). Describe any assumptions made about any missing or unclear information. | 3&6 |
| Study risk of bias assessment | 11 | Specify the methods used to assess risk of bias in the included studies, including details of the tool(s) used, how many reviewers assessed each study and whether they worked independently, and if applicable, details of automation tools used in the process. | 6-7 |
| Effect measures | 12 | Specify for each outcome the effect measure(s) (e.g., risk ratio, mean difference) used in the synthesis or presentation of results. | 3&6 |
| Synthesis methods | 13a | Describe the processes used to decide which studies were eligible for each synthesis (e.g., tabulating the study intervention characteristics and comparing against the planned groups for each synthesis (item #5)). | 6 |
|  | 13b | Describe any methods required to prepare the data for presentation or synthesis, such as handling of missing summary statistics, or data conversions. | 7&9-10 |
|  | 13c | Describe any methods used to tabulate or visually display results of individual studies and syntheses. | 3&6-7 |
|  | 13d | Describe any methods used to synthesise results and provide a rationale for the choice(s). If meta-analysis was performed, describe the model(s), method(s) to identify the presence and extent of statistical heterogeneity, and software package(s) used. | N/A |
|  | 13e | Describe any methods used to explore possible causes of heterogeneity among study results (e.g., subgroup analysis, meta-regression). | N/A |
|  | 13f | Describe any sensitivity analyses conducted to assess robustness of the synthesised results. | N/A |
| Reporting bias assessment | 14 | Describe any methods used to assess risk of bias due to missing results in a synthesis (arising from reporting biases). | 6-7 |
| Certainty assessment | 15 | Describe any methods used to assess certainty (or confidence) in the body of evidence for an outcome. | N/A |
| **RESULTS** | | |  |
| Study selection | 16a | Describe the results of the search and selection process, from the number of records identified in the search to the number of studies included in the review, ideally using a flow diagram. | 3&10-11 |
|  | 16b | Cite studies that might appear to meet the inclusion criteria, but which were excluded, and explain why they were excluded. | 3 |
| Study characteristics | 17 | Cite each included study and present its characteristics. | 4-5&10-11 |
| Risk of bias in studies | 18 | Present assessments of risk of bias for each included study. | S.M. 21 - 27 |
| Results of individual studies | 19 | For all outcomes, present, for each study: (a) summary statistics for each group (where appropriate) and (b) an effect estimate and its precision (e.g., confidence/credible interval), ideally using structured tables or plots. | 7-16 |
| Results of syntheses | 20a | For each synthesis, briefly summarise the characteristics and risk of bias among contributing studies. | S.M. 21 - 27 |
|  | 20b | Present results of all statistical syntheses conducted. If meta-analysis was done, present for each the summary estimate and its precision (e.g., confidence/credible interval) and measures of statistical heterogeneity. If comparing groups, describe the direction of the effect. | N/A |
|  | 20c | Present results of all investigations of possible causes of heterogeneity among study results. | N/A |
|  | 20d | Present results of all sensitivity analyses conducted to assess the robustness of the synthesised results. | N/A |
| Reporting biases | 21 | Present assessments of risk of bias due to missing results (arising from reporting biases) for each synthesis assessed. | S.M. 21 - 27 |
| Certainty of evidence | 22 | Present assessments of certainty (or confidence) in the body of evidence for each outcome assessed. | N/A |
| **DISCUSSION** | | |  |
| Discussion | 23a | Provide a general interpretation of the results in the context of other evidence. | 16-19 |
|  | 23b | Discuss any limitations of the evidence included in the review. | 17-19 |
|  | 23c | Discuss any limitations of the review processes used. | 19 |
|  | 23d | Discuss implications of the results for practice, policy, and future research. | 19-20 |
| **OTHER INFORMATION** | | |  |
| Registration and protocol | 24a | Provide registration information for the review, including register name and registration number, or state that the review was not registered. | 2 |
|  | 24b | Indicate where the review protocol can be accessed, or state that a protocol was not prepared. | 2 |
|  | 24c | Describe and explain any amendments to information provided at registration or in the protocol. | N/A |
| Support | 25 | Describe sources of financial or non-financial support for the review, and the role of the funders or sponsors in the review. | 20 |
| Competing interests | 26 | Declare any competing interests of review authors. | 20 |
| Availability of data, code and other materials | 27 | Report which of the following are publicly available and where they can be found: template data collection forms; data extracted from included studies; data used for all analyses; analytic code; any other materials used in the review. | NA |

# Sample characteristics: Race and ethnicity

Ethnicity or race was reported in 15 of 30 studies. Specifically, Supplementary Table 1 shows the race or ethnicity data that was available from each included study. We standardized this data into % Caucasian which was the most frequently reported metric and has been computed across studies for consistency.

**Supplementary Table 1:** Overview of ethnicity and/or race in cannabis and control groups

| **1st Author (Year)** | **Ethnicity** | | **Race** | | **%Caucasian** | |
| --- | --- | --- | --- | --- | --- | --- |
|  | **Cannabis** | **Controls** | **Cannabis** | **Controls** | **Cannabis** | **Controls** |
| Cousijn (2022) | - | - | - | - | - | - |
| Knodt (2022) | - | - | - | - | 93% | |
| Lichenstein (2022) | - | - | 51.3% European American  41.1% African American  7.6% Other races | | 51.3% | |
| Koenis (2021) | - | - | - | - | - | - |
| Manza (2020) | - | - | - | - | - | - |
| Sweigert (2020) | 3 Hispanic/Latino  23 Not Hispanic/Latino | 1 Hispanic/Latino  24 Not Hispanic/Latino | 21 Caucasian  1 Asian  1 African American  3 Other | 12 Caucasian  11 Asian  0 African American  2 Other | 80.8% | 48% |
| Levar (2018) | - | - | Cannabis and Controls matched on race | | - | - |
| Jakabek (2016) | - | - | - | - | - | - |
| Orr (2016) | 5.2% Hispanic/Latino  48.6% Not Hispanic/Latino  0.3% Unknown or not reported | 3.1% Hispanic/Latino  42.1% Not Hispanic/Latino  0.6% Unknown or not reported | 0.1% American Indian/Alaskan Native  2.9% Asian/Native Hawaiian/other Pacific Islander  9.7% Black or African American  1.5% More than one  1.3% Unknown/not reported  38.7% White | 0.1% American Indian/Alaskan Native  2.3% Asian/Native Hawaiian/other Pacific Islander  6.7% Black or African American  0.8% More than one  0.3% Unknown/not reported  35.5% White | 38.7% | 35.5% |
| Rigucci (2016) | 50% (18) White Caucasian  20% (7) Black Caribbean  22% (8) Black African  8% (3) Other | | - | - | 50% | |
| Yucel (2016) | - | - | - | - | - | - |
| Becker (2015) | 19 Caucasian  4 Other | 21 Caucasian  2 Other | - | - | 82.6% | 91.3% |
| Epstein (2015) | - | - | - | - | - | - |
| Shollenbarger (2015) | 33.33% Ethnic minority  66.7% Caucasian | 32.35% Ethnic minority  67.7% Caucasian | - | - | 66.7% | 67.7% |
| Epstein (2014) | 23/31 White | 46/55 White | - | - | 74.2% | 83.6% |
| Filbey (2014) | - | - | 25/62 White  27/62 Latino  5/62 Native American  4/62 Black  1/62 Asian | 24/48 White  16/48 Latino  3/48 Native American  3/48 Black  0/48 Asian | 40.3% | 50% |
| Gruber (2014) | - | - | - | - | - | - |
| Jacobus (2013a) | 65% Caucasian | 65% Caucasian | - | - | 65% | 65% |
| Jacobus (2013b) | 76% Caucasian | 56% Caucasian | - | - | 76% | 56% |
| Zalesky (2012) | - | - | - | - | - | - |
| Gruber (2011) | - | - | - | - | - | - |
| Kim (2011) | - | - | - | - | - | - |
| Yucel (2010) | - | - | - | - | - | - |
| Ashtari (2009) | 0 Caucasian  14 Non-Caucasian | 3 Caucasian  11 Non-Caucasian | - | - | 0% | 21.4% |
| Bava (2009; 2010) | 61.6% Caucasian | 62.9% Caucasian | - | - | 61.6% | 62.9% |
| Jacobus (2009) | 43% Caucasian | 57% Caucasian | - | - | 43% | 57% |
| Arnone (2008) | - | - | - | - | - | - |
| DeLisi (2006) | - | - | - | - | - | - |
| Gruber (2005) | - | - | - | - | - | - |

# Additional information on cannabis exposure metrics

This section overviews how cannabis exposure levels were measured. Specifically, it outlines how cannabis exposure levels were measured (e.g., age of onset, duration, dosage, frequency), how cannabis exposure levels were converted to standardised metrics, and the inclusion/exclusion criteria for cannabis exposure in cannabis and control groups.

## Overview of reporting of cannabis exposure metrics

This section entails an overview of which key cannabis exposure metrics were measured in the literature, including duration, age of onset, dosage, and frequency of use. The *duration of cannabis use* was reported by 16 studies all in years, with 1 exception which reported it in months. The *age of onset of cannabis use* was reported in almost all studies (n=22). It was measured inconsistently as shown in Supplementary Figure 1.


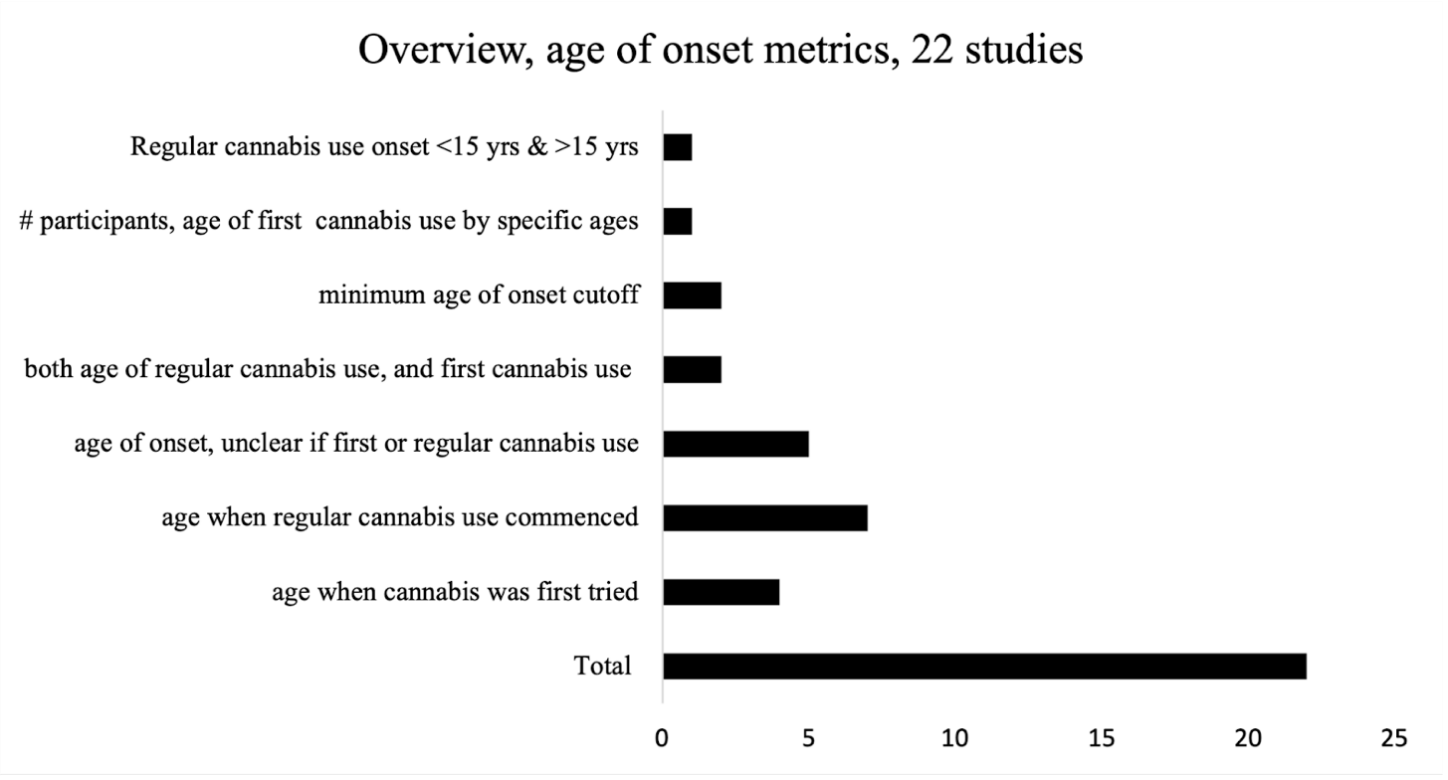
**Supplementary Figure 1.** Overview of metrics used for the age of onset of cannabis use

*Cannabis dosage* was measured in 15 studies using various measures (e.g., joints, grams, hits) and over different timeframes outlined in Supplementary Figure 2.


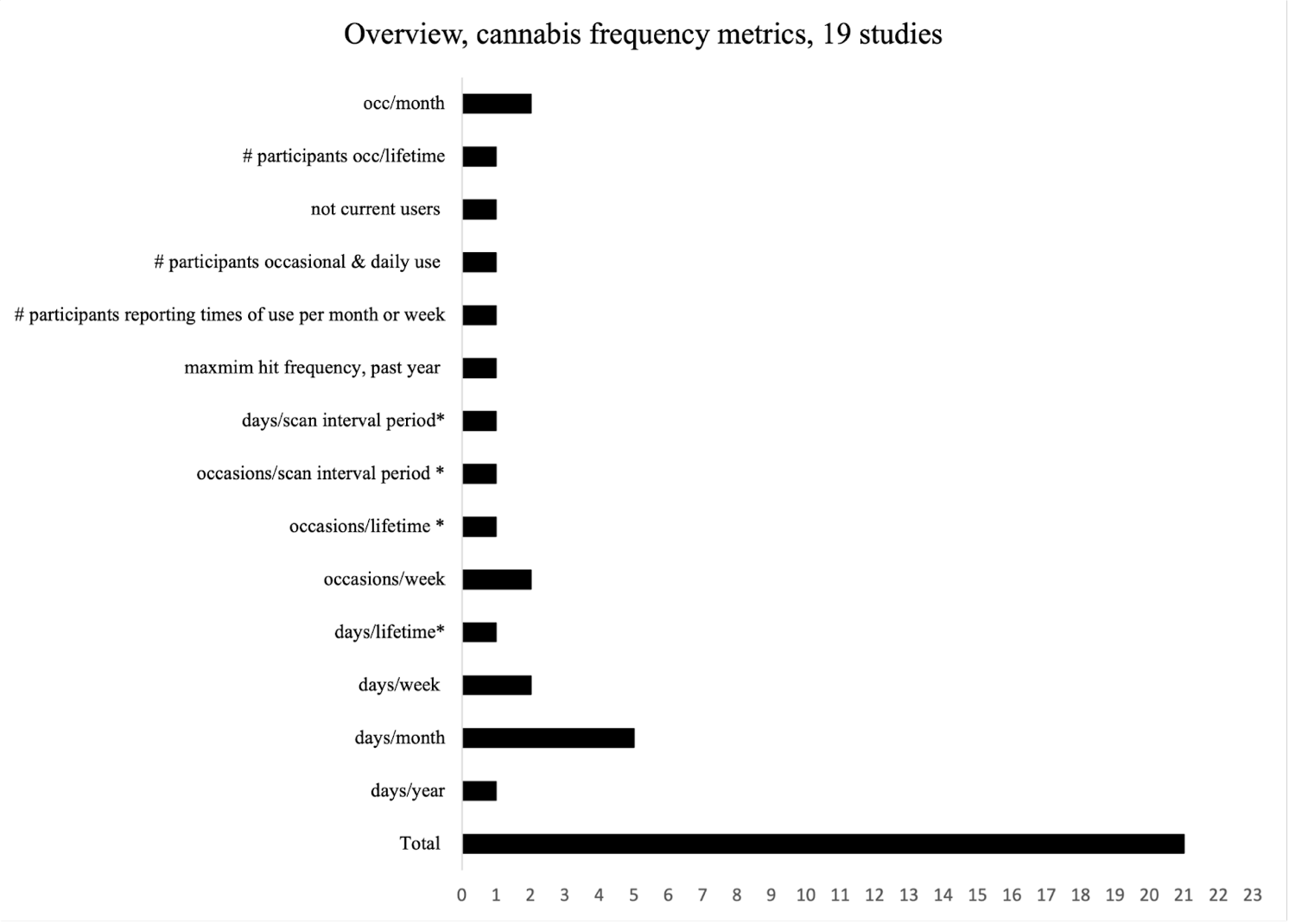
**Supplementary Figure 2.** Overview of metrics used for cannabis dosage

The *frequency of cannabis use* (Supplementary Figure 3) was measured by 15 studies using heterogeneous metrics (as referred to with ‘*’ in Figure 3), including 2 longitudinal studies that reported 2 measures of frequency of cannabis use, 1 prior to baseline testing and 1 between baseline and follow-up (Jacobus et al., 2013b; Epstein and Kumra, 2015).


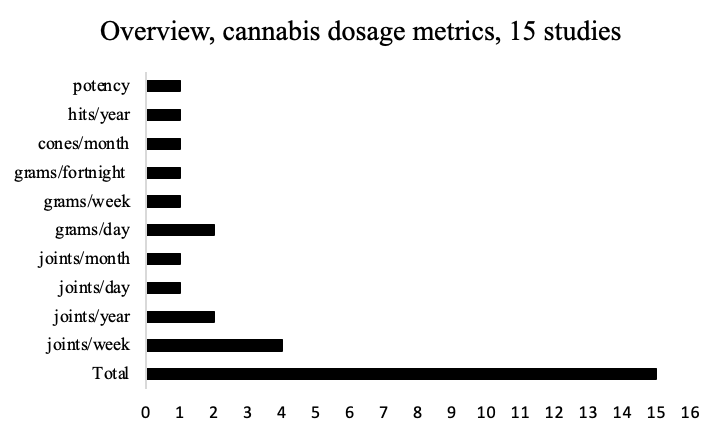
**Supplementary Figure 3.** Overview of metrics used for cannabis use frequency

## Conversion to standardised metrics

We standardised cannabis exposure metrics to enable systematic integration of the results. *Age of cannabis use onset* and *duration* of cannabis use were converted to years. *Cannabis dosage* was converted to ‘standard cones’/month. Specifically, cannabis dosage was converted to ‘cones’, whereby 1 joint = 3 cones, and 1 gram = 12 cones (Lorenzetti et al., 2016); and the timeframes given for dosage (e.g., per day, week, fortnight, month, year) were converted to monthly. Cannabis use *frequency* was converted to monthly. We reported ‘occasions’/month and ‘hits’/month separately as they could not be converted to standardised dosage measures used herein. In addition, we reported lifetime measures of exposure where other metrics were not reported, and over ‘inter-scan interval’ for longitudinal studies.

## Overview of inclusion and exclusion thresholds for cannabis exposure levels

Heterogeneous thresholds were used to include and exclude levels of cannabis exposure and related problems in the cannabis and control groups, as outlined below and in Supplementary Table 2.

### Thresholding cannabis exposure levels in the cannabis user groups

Cannabis users were included based on set criteria for cannabis use and problems with cannabis use. 26 studies set a minimum cut-off for cannabis exposure levels, using either 1 or multiple cannabis use metrics.

#### Minimum thresholds for frequency of cannabis use

Six studies required cannabis users to use for a minimum *frequency* (e.g., a certain number of days/occasions per week/month) (Gruber and Yurgelun-Todd, 2005; Bava et al., 2009; Jacobus et al., 2009; Levar et al., 2018; Sweigert et al., 2020; Lichenstein et al., 2022) and another required a minimum dosage over the last year, and during participants lifetime (Shollenbarger et al., 2015). Minimum occasions of use were required by 2 studies (Jacobus et al., 2013a; Jacobus et al., 2013b).

Nine studies required a minimum cannabis use *frequency* and also *duration* (Arnone et al., 2008; Yücel et al., 2010; Kim et al., 2011; Zalesky et al., 2012; Filbey et al., 2014; Jakabek et al., 2016; Yucel et al., 2016; Cousijn et al., 2022; Knodt et al., 2022). 1 study included participants with minimum cannabis use frequency and lifetime joints (Gruber et al., 2011).

**Supplementary Table 2:** Overview of inclusion and exclusion of cannabis exposure levels in cannabis and control groups

| **1^st^ Author (Year)** | **Cannabis Users** |  |  | **Controls** |  |
| --- | --- | --- | --- | --- | --- |
|  | **Included user type,**  **as per study description** | **Cannabis use level** | **Positive Urinary for THC metabolites** | **Cannabis use level** | **Positive Urinary for THC metabolites** |
| Cousijn (2022) | Weekly to daily user | Freq. ≥ 10 occ./month &  Durat.  ≥ 2 years | _ | 1-50 lifetime occ. & no use past year | _ |
| Knodt (2022) | Long-term or dependent user | Freq. ≥ once/week  Durat. ≥ 1 year,  OR Cannabis Dependence & Freq. ≥ once/week, at ≥ 1 study wave | _ | Never used (also no illicit drug dependence) | _ |
| Lichtenstein (2022) | Moderate or heavy user | Freq. ≥ once/week | _ | ≤1 day/month | _ |
| Koenis (2021) | DSM-5 CUD current or remitted | _ | Inclusion criterion | No use | Excluded |
| Manza (2020) | Lifetime DSM-IV Cannabis Dependence - DSM - IV | _ | Not excluded | <10 lifetime use | _ |
| Sweigert (2020) | At risk for CUD | Freq. ≥ once/week | _ | Never used | _ |
| Levar (2018) | Use | Freq. ≥ once/week | _ | < 5 lifetime occ. & no use past 6 months | _ |
| Jakabek (2016) | Regular Use | Freq. ≥ twice/month &  Durat.  ≥3 years | Not excluded | _ | _ |
| Orr (2016) | Recreational user | Dosage >100 occ. lifetime &  Age of onset < 18 | _ | Never used (no lifetime use) | _ |
| Rigucci (2016) | High/low potency use | Occ. & daily use at any point during lifetime | _ | Never used (no lifetime use) | _ |
| Yucel (2016) | Long-term & regular user | Freq. ≥ twice/ month &  Duration ≥2 years | Inclusion criterion | Never used | Excluded |
| Becker (2015) | Regular user | Freq. ≥5 times/week,  Durat. ≥ 1 year &  Age of onset <17 | _ | ≤5 occ., past year | _ |
| Epstein (2015) | CUD treatment seeking | Dosage > 50 occ. prior to age 17 | Not excluded | < 5 lifetime occ. (all illicit drugs) | All negative |
| Shollenbarger (2015) | Chronic user | Dosage lifetime > 50 joints & dosage past year > 25 joints | _ | ≤ 5 occ. past year & 10 lifetime joints. | _ |
| Epstein (2014) | Significant user | Dosage > 50 occ.  prior to age 17 | Not excluded | < 5 lifetime occ. (all illicit drugs) | All negative |
| Filbey (2014) | Regular user | Freq. ≥ 4 times/week,  over past 6 months | Inclusion criterion | No regular use | All negative |
| Gruber (2014) | Chronic, heavy user | Freq. ≥ 4 days/past week &  Dosage ≥ 2,500 occ. | Not excluded | <15 lifetime occ. (also of any category of illicit drugs) | Excluded |
| Jacobus (2013a) | Heavy user | Dosage > 200 lifetime occ. | _ | < 10 lifetime use occ. | _ |
| Jacobus (2013b) | Substance user (cannabis and alcohol) | Dosage > 200 lifetime occ. | Exclusion criterion to confirm ≥ 28 day abstinence | < 10 lifetime use occ. | _ |
| Zalesky (2012) | Long-term heavy user | Freq. ≥ 2 times/month,  Durat. ≥ 3 years | Not Excluded | average cumulative lifetime dose of 8 joints, & 1 joint in past year* | _ |
| Gruber (2011) | Chronic, heavy user | Freq. ≥ 4 days/past week &  Dosage ≥ 3,000 lifetime joints | Not excluded | ≤ 10 lifetime occ. | All negative |
| Kim (2011) | Heavy user | Freq. ≥ once/week, over the past month | Required to confirm self-report | Never use (& never use for any illicit drugs) | All negative |
| Yucel (2010) | Long-term user | Freq. ≥ once/week &  duration ≥ 1 year | _ | Never use (& never abuse for any illicit drugs) | _ |
| Ashtari (2009) | DSM-IV Dependence in remission in Residential Rehabitation | Freq. daily use, for duration of ≥ 1 year prior to treatment | Excluded criterion to confirm ≥ 3 months abstinence | ≤ 5 lifetime occ. (& to other illicit drugs) | _ |
| Bava (2009;2010) | Heavy user  (with heavy alcohol use) | Dosage ≥ 180 lifetime occ. | Exclusion criterion | 1.4 lifetime occ.* | _ |
| Jacobus (2009) | Heavy user  (with binge drinking) | Dosage ≥ 180 life occ. | Exclusion criterion to confirm ≥ 23 day abstinence | ≤ 5 lifetime occ. | All negative |
| Arnone (2008) | Heavy user, early adolescent onset | Freq. Daily use for ≥ 2 years | Inclusion criterion | _ | Excluded |
| DeLisi (2006) | Current user | Dosage >21 occ. in any single year & Age of onset <18 | _ | Never use (& never use for any illicit drugs) | _ |
| Gruber (2005) | Heavy user | Dosage ≥ 4000 lifetime occ. | Inclusion criterion | ≤ 3 lifetime occ. | Excluded |

*Note.* DSM = diagnostic and statistical manual for mental disorders, Durat. = duration,  Freq. = frequency, Occ.  = occasions, THC = delta-9-tetrahydrocannabinol; * reported in results section, not as part of inclusion/exclusion criteria.

#### Problems with cannabis use

Only 5 studies included participants with *cannabis dependence or abuse*, as per DSM-IV. Cannabis dependence (current or in remission) was an inclusion criterion in 2 studies (Manza et al., 2020; Koenis et al., 2021). In addition, 1 study (Knodt et al., 2022) set an inclusion criterion of *long-term or dependent cannabis users*. Disordered cannabis use was an inclusion criterion together with minimum cutoffs for occasions of use and adolescent age of onset (Epstein and Kumra, 2015), occasions of use and either frequency (Gruber et al., 2014) or duration (Ashtari et al., 2009). Lastly, a minimum score of ≥ 4 for Cannabis Use Disorder was a required inclusion criterion as per Alcohol Smoking and Substance Involvement Screening Test (Sweigert et al., 2020).

#### Minimum age of cannabis use onset and others

Specific a*ges of cannabis use onset* was an inclusion criterion in 4 studies, combined with selected occasions of use (Delisi et al., 2006; Epstein et al., 2014), frequency and duration (Becker et al., 2015), and minimum cannabis use occasions (Orr et al., 2016).

#### Urinary Cannabinoids

The level of cannabinoids or cannabinoid metabolites was measured in 15 studies. Some studies required a negative urinary cannabinoid test to confirm participants’ self-reported abstinence from cannabis (n = 4 (Ashtari et al., 2009; Bava et al., 2009; Jacobus et al., 2009; Jacobus et al., 2013b)), while in others the presence of THC metabolites was required for inclusion to confirm current cannabis use (Gruber and Yurgelun-Todd, 2005; Arnone et al., 2008; Filbey et al., 2014). One study also used urinary cannabinoid levels to categorise users into subgroups and to verify self-reported substance use (THC and CBD), also incorporating a former user group (all reporting negative for urinary cannabinoids) (Yucel et al., 2016). Eight studies included measures of urinary cannabinoids in their cannabis users, however, users were either not excluded if they tested positive, or positive results was not clearly an exclusion/inclusion criterion for participants in these studies.

### Controls

In control groups, 15 studies excluded controls based on distinct cannabis exposure levels: non-cannabis users (3 studies) (Yucel et al., 2016; Sweigert et al., 2020; Koenis et al., 2021); no lifetime history of cannabis use or any substance use (6 studies) (Delisi et al., 2006; Yücel et al., 2010; Kim et al., 2011; Orr et al., 2016; Rigucci et al., 2016; Knodt et al., 2022), or did not clearly define cannabis use levels in their control groups (5 studies).

# Overview of diffusion-weighted MRI Acquisition tools and Parameters

The reviewed studies used distinct tools and parameters to acquire diffusion-MRI data (Supplementary Table 3). All studies reported *scanner strength*, which was reported as 3T in 25 studies. All studies reported the *b-values of diffusion-weighted volumes*, which were most commonly 1000 s/mm^2^ (15 studies, range: 600-to-3000 s/mm^2^); as well as *echo time* (range = 66-to-110 ms); and *b-value=0*, which were most commonly single values (n=23). The majority of studies reported additional parameters, such as *repetition time* (range: 2400-to-14000 ms) and *voxel size* (range: 1.25-4 millimeters), *field of view* and the number of *b = 0 non-diffusion weighted volumes* (range: 1-10, but most commonly 1).

About half the studies reported additional parameters including the *number of slices* (range: 19-to-75); *image matrix, number of scan directions* (range = 6-to-90), *acquisition plane* (largely axial); slice *thickness* (range: 2-to-5 mm); and the *total number of volumes* acquired during scans (range: 4-to-42 volumes). Only a few studies reported additional parameters: *scan duration* (range: 3-to-18 minutes); the *number of images* acquired (range: 32-to-45); as well as *pulse sequence*, which was heterogeneous, and cardiac gating (2 studies).

**Supplementary Table 3:** Acquisition parameters for diffusion-weighted MRI scans

|  | Whole Brain/  ROI | MR Strength | Pulse Sequence | TE,  *ms* | TR,  *ms* | FOV,  *mm^3^* | Voxel size, *mm* | Matrix | Plane | Slices,  *N* | Slice Thick,  *mm* | Directions,  *N* | Duration,  *mins* | Vols,  *N* | b value  =0 | b value  s/mm^2^ |
| --- | --- | --- | --- | --- | --- | --- | --- | --- | --- | --- | --- | --- | --- | --- | --- | --- |
| Cousijn (2022) | whole brain | 3T | Pulse gradient, spin-echo EPI | 71 | 7329 | 240×240×150 | 1.875 | _ | _ | 75 | 2 | _ | _ | 30 | 1 | 1000 |
| Knodt (2022) | whole brain | 3T | _ | 110 | 4700 | 240 | 2.5 | 96x96 | _ | _ | 2.5 | _ | _ | _ | 2 | 3000 |
| Lichenstein (2022) | ROI: ATR, cingulum | 3T | _ | 91 | 8400 | 256x256 | _ | 96x96 | Axial | 64 | 2 | 61 | 9:56 | _ | 7 | 1000 |
| Manza (2020) | whole brain | 3T | _ | 89 | 5500 | _ | 1.25 | _ | _ | _ | _ | 90 | _ | _ | 6 | 1000, 2000, 3000 |
| Koenis (2021) | whole brain | 3T | _ | 92.8 | 4250 | _ | 1.8 | _ | _ | _ | _ | 90 | 6:50 | _ | 1 | 2000 |
| Sweigert (2020) | ROI: cerebellar peduncles, pontine | 3T | Pulsed-gradient spin-echo | 75 | 8500 | 256x256x150 | 2x2x2 |  | Axial | _ | _ | _ | 8:25 | 32 | 4 | 1000 |
| Levar (2018) | ROI: uncinate fasciculus | 3T | Single spin-echo EPI | 66 | 2400 | 256 | 2 | _ | _ | _ | _ | 60 | 3:24 | _ | 10 | 900, 2000 |
| Jakabek (2016) | whole brain | 3T | Spin-echo EPI | 96 | 7000 | 240×240 | 2.3×2.3 | _ | _ | 54 | 2.3 | _ | _ | _ | 5 | 2000 |
| Orr (2016) | whole brain | 3T | Spin-echo EPI | 89 | 5500 | _ | 1.25 | _ | _ | _ | _ | 90 | _ | _ | 6 | 1000, 2000, 3000 |
| Rigucci (2016) | ROI: corpus callosum | 3T | Dual spin-echo EPI | 104.5 | _ | _ | 2.4×2.4×2.4 | _ | Near axial | 60 | _ | _ | _ | _ | 4 | 1300 |
| Yucel (2016) | ROI: fimbria, cingulum (hipp. portion) | 3T | Spin-echo EPI | 96 | 7000 | 240×240 | 2.3×2.3 | 104×104 | Axial | 54 | 2.3 | _ | _ | 42 | _ | 2000 |
| Becker (2015) | whole brain | 3T | Dual spin echo, single-shot, pulsed gradient EPI | 90 | 8500 | 256 | 2×2×2 | _ | Axial | 64 | _ | _ | _ | 30 | 6 | 1000 |
| Epstein (2015) | whole brain | 3T | Dual spin echo, single-shot, pulsed gradient EPI | 98 | 8500 | 256 | 2×2×2 | _ | Axial | 64 | _ | _ | _ | 30 | 6 | 1000 |
| Shollenbarger (2015) | ROI: frontolimbic | 4T | _ | 88.8 | 8000 | 256 | 4×4×4 | 64×64×30 | _ | _ | _ | 12 | _ | _ | _ | 600 |
| Epstein (2014) | whole brain | 3T | Dual spin echo, single-shot, pulsed gradient EPI | 98 | 8500 | 256×256 | 2×2×2 | _ | Sagittal | 64 | _ | _ | _ | 30 | 6 | 1000 |
| Filbey (2014) | whole brain | 3T | Twice-refocused spin echo | 84 | 9000 | 256×256×144 | 2 × 2 × 2 | _ | _ | _ | _ | _ | _ | 30 | 5 | 800 |
| Gruber (2014) | whole brain | 3T | Dual spin echo, single-shot EPI | 89 | 9300 | 256 | _ | 128×128 | Axial | _ | 2 | 48 | 8:41 | _ | 7 | 700 |
| Jacobus (2013a) | whole brain | 3T | Dual spin-echo single shot EPI | 93 | 12000 | 240 | _ | 128×128 | _ | 36 | 3 | 15 | _ | 4 | _ | 2000 |
| Jacobus (2013b) | ROI: fornix, superior corona radiata, SLF, SFOF | 3 | Single-shot dual spin echo | 93.4 | 12400 | 240x240 | 1.875x1.875x3 | 128x128 | _ | 36 | _ | 15 | 13:39 | _ | _ | 2000 |
| Zalesky (2012) | whole brain | 3T | Spin echo EPI_ | 106 | 7400 | 240 × 240 | 2.3 × 2.3 | 104×104 | Axial | 54 | 2.3 | _ | _ | 42 | 5 | 2000 |
| Gruber (2011) | ROI: frontal, genu, splenium | 3T | Dual spin echo, single-shot EPI | 81 | 5000 | 210 | _ | 128×128 | Axial | _ | 5 | 6 | _ | _ | _ | 1000 |
| Kim (2011) | whole brain | 3T | Dual spin echo, single-shot EPI | 93 | 6500 | 230×230×144 | 1.8×1.8 ×3 | 128×128 | Axial | 48 | _ | _ | 9:10 | 20 | 1 | 1000 |
| Yucel (2010) | whole brain | 3T | Spin-echo EPI | 106 | 6100 | 220×220 | 1.72×1.72×3 | 128×128 | Axial | 38 | 3 | 48 | _ | _ | _ | 1000 |
| Ashtari (2009) | whole brain & ROI: SLF & motor | 1.5 | Dual spin-echo, single-shot EPI | 77 | 14000 | 220×220 | 2.5×2.5×2.5 | 88×88 | _ | 50 | 2.5 | 15 | 8:24 | _ | 2 | 1000 |
| Bava (2009;2010) | whole brain | 3T | Dual spin-echo single shot EPI | 93 | 12000 | 240 | _ | 128×128 | _ | 36 | 3 | 15 | _ | _ | 1 | 2000 |
| Jacobus (2009) | whole brain | 3T | Dual spin-echo single shot EPI | 93.4 | 12400 | 240 | _ | 128×128 | _ | _ | 3 | 15 | _ | 4 | 1 | 2000 |
| Arnone (2008) | ROI: corpus callosum | 1.5T | Spin-echo EPI | _ | _ | 240×240 | 2.5 | 96×96 | _ | 50 | 2.8 | 12 | _ | _ | 1 | 1000 |
| DeLisi (2006) | ROI: temporal lobe, STG, hippocampus, amygdala | 1.5T | _ | 100 | 6000 | _ | 2.5×2.5 | 128×128 | _ | 19 | 5 | 8 | _ | _ | 1 | 1000 |
| Gruber (2005) | ROI: ACC, DLPFC | 3T | Twice-refocused spin echo | 83 | 4500 | 200 | _ | 128×128 | Axial | _ | 5 | _ | _ | _ | 1 | 1000 |

*Note.* ACC = anterior cingulate cortex, DLPFC = dorsolateral prefrontal cortex, EPI = Echo Planar Imaging, Hipp. = Hippocampal, ROI = region of interest, Sag. = sagittal, STG = superior temporal gyrus, T = Tesla, TE = Echo Time, TR = Repetition Time, FOV = Field of View. For Manza and colleagues (2020), additional data on the acquisition parameters from the human connectome project were taken from an additional source (Ugurbil et al., 2013). N images was given by Levar and colleagues (2018), Jakabek and colleagues (2016) and Rigucci and colleagues (2016) and it was 45, 42 and 32, respectively. Cardiac gating was measured by 2 studies, Cousijn and colleagues (2022) and Rigucci and colleagues (2016).

# Overview of emerging group differences in white matter’ fractional anisotropy, mean diffusivity and other diffusion-MRI metrics, reported by up to 3 studies

This section describes group differences in fractional anisotropy (FA), mean diffusivity (MD), and other diffusion-MRI metrics that were detected within the research, but which were not consistent across the literature.

## FA differences

### FA differences in ≤ 3 studies

Three studies each found lower FA in additional white matter fiber tracts: the inferior fronto-occipital fasciculus (Jacobus et al., 2013a; Epstein et al., 2014; Koenis et al., 2021), uncinate fasciculus (Jacobus et al., 2013a; Shollenbarger et al., 2015; Manza et al., 2020), and tracts within the temporal lobe (Ashtari et al., 2009; Bava et al., 2009; Manza et al., 2020). Two studies each found lower FA in the anterior thalamic radiations (Jacobus et al., 2013; Koenis et al., 2021), with 1 study also showing higher FA (Lichenstein et al., 2022).

### FA differences in ≤ 2 studies

Two studies also found lower FA in the external capsule (Gruber et al., 2014; Manza et al., 2020), corona radiata (Jacobus et al., 2013a; Manza et al., 2020), and frontal lobe based tracts (Bava et al., 2009; Gruber et al., 2011). Additionally, higher, and lower FA was observed in 2 separate studies respectively in the forceps *minor* (Filbey et al., 2014; Jakabek et al., 2016). Separate studies also showed both higher and lower FA in the parietal lobe, with lower FA for white matter tracts within the left postcentral gyrus (Bava et al., 2009), and higher FA for tracts in the inferior parietal lobe (Delisi et al., 2006).

### FA differences in single studies

Single studies also found lower FA in the forceps *major* (Koenis et al., 2021), arcuate tract (Ashtari et al., 2009), midbrain, cerebellar peduncle (Jacobus et al., 2009), tracts adjacent to the hippocampus (Yücel et al., 2010), the temporo-thalamic tract, undisclosed occipito-frontal white matter regions (Bava et al., 2009), as well as the inferior longitudinal fasciculus (Epstein et al., 2014). A single study also showed higher FA in the cingulate, and frontal lobes (Delisi et al., 2006). Another also found higher FA in the occipital cuneus (Bava et al., 2009). Finally, 1 study found higher FA in the middle cerebellar peduncle in cannabis users compared to controls (Sweigert et al., 2020).

## Group differences in MD and RD, in single studies

There were multiple studies that implicated single tract differences in MD in cannabis users compared to controls. 1 study found higher MD in tracts within the occipital lobe, adjacent to the lingual gyrus (Bava et al., 2009). A single study also found higher MD in the arcuate, internal capsule, and temporal and occipital lobes (Ashtari et al., 2009), and the anterior thalamic radiation and uncinate fasciculus (Shollenbarger et al., 2015). Single studies also reported lower MD in the inferior longitudinal fasciculus (Bava et al., 2009), and the frontal lobe and the cingulate (Delisi et al., 2006). Finally, 1 study found lower MD and RD in the middle cerebellar peduncle in cannabis users compared to controls (Sweigert et al., 2020).

## Group differences in other metrics in single studies

A series of single studies reported group differences in other diffusion-MRI metrics. A single paper used graph theoretical analysis to evaluate differences in white matter microstructure in cannabis users versus controls (Kim et al., 2011). Increased normalised clustering coefficients, and decreased global efficiency was found in structural brain networks. The number of connections in the network matrix was also significantly decreased. Increased betweenness centrality was found in cingulate regions in cannabis user networks (p < 0.05, FDR corrected). However, no significant difference was found between groups regarding mean weights in the network matrix, and no difference in normalised characteristic path length, small worldness, or local efficiency of brain networks. Overall, this indicates heterogeneous findings in the white matter’s efficiency as a network in cannabis users compared to controls.

In addition, 1 paper used a novel network-based statistic to investigate white matter microstructure, in terms of differences in the number of streamlines in the whole brain, or various networks or tracts (Zalesky et al., 2012). For example, results found that the fimbria network showed an 84% reduction in cannabis users, 7 streamlines in cannabis users, and 44 in controls. A commissural fibre (originating at the corpus callosum (splenium) and ending within the precuneus) showed an 88% reduction in streamlines, 9 in cannabis users, and 78 in controls.

Levar and colleagues (2018) used both diffusion-MRI metrics (as reported in Tables 4, 5, 6 and 7 in the main manuscript), as well as bundle length, to investigate white matter microstructure in the uncinate fasciculus. The study found that bundle length in the uncinate fasciculus was significantly reduced in cannabis users compared to controls. This indicates poorer white matter integrity of this tract in cannabis users compared to controls.

# Additional emerging correlations between white matter and behavioral variables reported by ≤ 3 studies

This section describes studies that analysed correlations between white matter and indices of cannabis use, cognition, alcohol use, and mental health-related variables in no more than 3 studies. These findings indicate (1) the lack of consistency in running correlations for these metrics and (2) the lack of associations detected. Overall, the findings discussed in this section are not consistent throughout the literature, but demonstrate a lack of consistency in brain-behaviour correlations in cannabis use research.

## Cannabis exposure metrics

### Age of cannabis use onset

There were no significant correlations between cannabis exposure metrics associated with graphs theoretical analysis or fibre bundle length, and age of onset. As discussed in the main document of the manuscript, correlations between age of onset and diffusion-MRI metrics were related to FA, MD, RD, and AD. In addition to the consistent findings in the corpus callosum and ILF for FA, additional single white matter tracts were also implicated in these relationships. More specifically, earlier age of onset of cannabis use was positively correlated with FA in various other areas, including frontal regions (Gruber et al., 2011), SLF, lateral prefrontal white matter, and UF (Cousijn et al., 2022).

### Cannabis dosage

Three studies investigated the correlation between cannabis dosage and FA. However, only 1 showed significance, with FA in the cingulate gyrus positively correlated with current dose, and lower FA in the forceps minor associated with current dose (Jakabek et al., 2016). Two studies investigated correlations between MD and dosage, and 3 studies investigated the correlations between RD, AD and cannabis dosage, however, none indicated significant correlations. Overall, there is no consistent finding in the relationship between cannabis dosage and diffusion-MRI metrics.

### Cannabis use frequency

First, 2 studies investigated the correlation between FA and frequency of cannabis use (Jacobus et al., 2009; Lichenstein et al., 2022). The number of cannabis hits in the past 3 months was positively correlated with FA in the SLF, and lifetime occasions of cannabis use were positively correlated with FA in the superior corona radiata. Second, another study investigated associations with MD, RD and AD and frequency of cannabis use (Rigucci et al., 2016). Every-day cannabis users showed significantly higher total MD and AD in the corpus callosum than both occasional users and those who had never used cannabis. Additionally, daily high-potency cannabis users had a significantly higher total MD, RD, and AD, in the corpus callosum than both those who used low-potency cannabis daily and those who had never used cannabis or used cannabis weekly. Finally, 1 study that used graph theoretical analysis, found that normalised clustering coefficient, normalised characteristic path length, small-worldness, global efficiency, and mean local efficiency were not significantly correlated with frequency of cannabis use, or estimated lifetime use (Kim et al., 2011).

### Abstinence from cannabis use

A single study investigated the correlation between duration of abstinence from cannabis use and white matter microstructure (FA, MD, RD and AD), but did not find significant correlations (Ashtari et al., 2009).

### Severity of cannabis use disorder & Persistence of dependence/regular use

One study investigated the correlation between severity of cannabis use disorder (DSM-5 symptom count) and white matter differences (FA, MD, RD and AD), but did not find significant correlations (Cousijn et al., 2022). One study also investigated associations between persistence of dependence and FA, but did not show any significant findings (Knodt et al., 2022). However, persistent regular users did show decreased global average FA, as compared to less persistent users (Knodt et al., 2022).

### Cannabis cravings

One study investigated the correlation between cannabis craving scores and white matter characterisation (Sweigert et al., 2020) across FA, MD, RD, and AD. This study found a (1) positive correlation between FA and cravings scores and (2) negative correlation between MD, RD, and AD, and cravings scores. This suggests that higher cannabis cravings are correlated with decreased white matter integrity.

### Cognition, alcohol use, and mental health-related variables

#### Impulsivity

Overall, 2 studies measured correlations between Barratt Impulsivity Scores (BIS) and white matter integrity, using FA. Gruber and colleagues (2014) found a significant negative correlation between FA in the left and right genu, and BIS attention, and also between FA in the right genu and motor scores. Gruber and colleagues (2011) also showed a significant positive relationship between FA in the left frontal region and BIS Total and Motor subscale scores. FA in the right frontal region was also significantly associated with BIS Total and Attention scores. More specifically, this indicated a positive correlation between FA in the frontal regions bilaterally and higher levels of impulsivity for cannabis users.

Using graph theoretical analysis, correlations between subscales of the BIS and white matter integrity were measured. Kim and colleagues (2011) found that global efficiency was significantly positively correlated with BIS subscales; Attention, Motor, and Non-Planning in cannabis users.

#### Neurocognitive Performance

A single study found there was no significant relationship between global neurocognitive performance and white matter differences (Jacobus et al., 2013a).

#### Memory

A single study investigated the correlation between fibre bundle length and memory performance as measured by the long-delay cued recall in the California Verbal Learning Task, however, this was not significant (Levar et al., 2018).

#### Alcohol Use

A single study, using graph theoretical analysis, found that global efficiency was significantly correlated with drinks per week in cannabis users (Kim et al., 2011).

#### Mental Health (Symptoms of Depression and Apathy)

One study showed that greater symptoms of depression were associated with lower FA in the anterior thalamic radiation bilaterally (Shollenbarger et al., 2015). Higher self-reported apathy symptoms were also associated with lower FA in the uncinate fasciculus bilaterally. Greater symptoms of depression were associated with higher MD in the left anterior thalamic radiation.

# Overview of Risk of Bias assessment

The results of the risk of bias analysis are as outlined in Supplementary Table 4. Overall, the quality of the evidence was assessed across all criteria per study, across all studies per criterion, and across all studies and criteria. This was done via computing the mean ratings, whereby 0 represented the lack of endorsement of criterion and hence high risk of bias; while 1 represented the endorsement of each criterion and low risk of bias.

Overall, the reviewed work had a moderate average rating of 0.7 out of 1 out of all studies across all criteria, indicating moderately low risk of bias. The quality rating of individual studies assessed against all criteria was high in 2 studies (i.e., 0.8-to-0.9 out of 1), moderately high in 9 studies (i.e., 0.7 out of 1 in 9 studies) and moderate in most studies (i.e., 0.5-to-0.6 out of 1). Meanwhile, the quality of the literature across each criterion was high for more than half of the criteria (i.e., 0.8-to-1 out of 1 for 9 criteria); moderately high for 1 criterion (i.e., 0.6 out of 1); low for the remaining 4 criteria (i.e., 0 out of 1); and not applicable for 1 criterion (i.e., blinding). The section below overviews further information about which items received high, moderate, and low risk of bias ratings.

**Supplementary Table 4: Overview of the Risk of Bias using the Quality Assessment Tool for Observational Cohort and Cross-Sectional Studies**

| **criteria** | **objective** | **population** | **partic. rate** | **same population/**  **period** | **uniform inc/ex criteria** | **power** | **i.v. measured prior to d.v.** | **time i.v. & d.v.** | **Corr./**  **subgroup analyses** | **i.v. defined** | **i.v. dependent measures** | **d.v. defined** | **blind** | **loss 20% FU** | **confounder** | ***avg. rating all criteria per study*** |
| --- | --- | --- | --- | --- | --- | --- | --- | --- | --- | --- | --- | --- | --- | --- | --- | --- |
|  | **I** | **II** | **III** | **IVa** | **IVb** | **V** | **VI** | **VII** | **VIII** | **IX** | **X** | **XI** | **XII** | **XIII** | **XIV** |  |
| **avg. rating all studies per criterion** | **1** | **1** | **0** | **0.8** | **1** | **0.1** | **0** | **0.8** | **0.6** | **1** | **0.2** | **1** |  | **1** | **0.8** | ***0.7*** |
| Cousijn (2022) | 1 | 1 | 0 | 1 | 1 | 0 | 0 | 1 | 1 | 1 | 0 | 1 |  |  | 1 | ***0.7*** |
| Knodt (2022) | 1 | 1 | 0 | 1 | 1 | 1 | 0 | 0 | 0 | 0 | 1 | 1 |  |  | 1 | ***0.6*** |
| Liechtenstein (2022) | 1 | 1 | 0 | 1 | 1 | 0 | 0 | 1 | 1 | 1 | 1 | 1 |  | 1 | 1 | ***0.8*** |
| Koenis (2021) | 1 | 1 | 0 | 1 | 1 | 0 | 0 | 1 | 0 | 1 | 0 | 1 |  |  | 1 | ***0.6*** |
| Manza (2020) | 1 | 1 | 0 | 1 | 1 | 1 | 0 | 0 | 0 | 1 | 0 | 1 |  |  | 0 | ***0.5*** |
| Sweigert (2020) | 1 | 1 | 0 | 1 | 1 | 0 | 0 | 1 | 0 | 1 | 0 | 1 |  |  | 1 | ***0.6*** |
| Levar (2018) | 1 | 1 | 0 | 0 | 1 | 0 | 0 | 1 | 0 | 1 | 0 | 1 |  |  | 1 | ***0.5*** |
| Jakabek (2016) | 1 | 1 | 0 | 1 | 1 | 0 | 0 | 1 | 1 | 1 | 0 | 1 |  |  | 1 | ***0.7*** |
| Orr (2016) | 1 | 1 | 0 | 1 | 1 | 0 | 0 | 0 | 1 | 1 | 0 | 1 |  |  | 1 | ***0.6*** |
| Rigucci (2016) | 1 | 1 | 0 | 1 | 1 | 0 | 0 | 1 | 1 | 1 | 0 | _ |  |  | 1 | ***0.7*** |
| Yücel (2016) | 1 | 1 | 0 | 0 | 1 | 0 | 0 | 1 | 0 | 1 | 0 | 1 |  |  | 1 | ***0.5*** |
| Becker (2015) | 1 | 1 | 0 | 0 | 1 | 0 | 0 | 1 | 0 | 1 | 1 | 1 |  | 1 | 1 | ***0.6*** |
| Epstein (2015) | 1 | 1 | 0 | 0 | 1 | 0 | 0 | 1 | 0 | 1 | 1 | 1 |  | 1 | 1 | ***0.6*** |
| Shollenbarger (2015) | 1 | 1 | 1 | 1 | 1 | 0 | 0 | 1 | 1 | 1 | 0 | 1 |  |  | 1 | ***0.8*** |
| Epstein (2014) | 1 | 1 | 0 | 0 | 1 | 0 | 0 | 0 | 0 | 1 | 0 | 1 |  |  | 1 | ***0.5*** |
| Filbey (2014) | 1 | 1 | 0 | 1 | 1 | 0 | 0 | 1 | 1 | 1 | 0 | 1 |  |  | 1 | ***0.7*** |
| Gruber (2014) | 1 | 1 | 0 | 1 | 1 | 0 | 0 | 1 | 1 | 1 | 0 | 1 |  |  | 0 | ***0.6*** |
| Jacobus (2013a) | 1 | 1 | 0 | 1 | 1 | 0 | 0 | 1 | 1 | 1 | 1 | 1 |  | 1 | 0 | ***0.7*** |
| Jacobus (2013b) | 1 | 1 | 0 | 1 | 1 | 0 | 0 | 0 | 0 | 1 | 1 | 1 |  | 1 | 1 | ***0.6*** |
| Zalesky (2012) | 1 | 1 | 0 | 1 | 1 | 0 | 0 | 1 | 1 | 1 | 0 | 1 | _ | _ | 1 | ***0.7*** |
| Gruber (2011) | 1 | 1 | 0 | 1 | 1 | 0 | 0 | 1 | 1 | 1 | 0 | 1 |  |  | 0 | ***0.6*** |
| Kim (2011) | 1 | 1 | 0 | 1 | 1 | 0 | 0 | 1 | 0 | 1 | 0 | 1 |  |  | 1 | ***0.6*** |
| Yücel (2010) | 1 | 1 | 0 | 1 | 1 | 0 | 0 | 1 | 1 | 1 | 0 | 1 |  |  | 1 | ***0.7*** |
| Ashtari (2009) | 1 | 1 | 0 | 0 | 1 | 0 | 0 | 1 | 1 | 1 | 0 | 1 |  |  | 1 | ***0.6*** |
| Bava (2009;2010) | 1 | 1 | 0 | 1 | 1 | 0 | 0 | 1 | 1 | 1 | 0 | 1 |  |  | 1 | ***0.7*** |
| Jacobus (2009) | 1 | 1 | 0 | 1 | 1 | 0 | 0 | 0 | 1 | 1 | 0 | 1 |  |  | 1 | ***0.6*** |
| Arnone (2008) | 1 | 1 | 0 | 1 | 1 | 0 | 0 | 1 | 1 | 1 | 0 | 1 |  |  | 1 | ***0.7*** |
| DeLisi (2006) | 1 | 1 | 0 | 1 | 1 | 0 | 0 | 0 | 0 | 1 | 0 | 1 |  |  | 1 | ***0.5*** |
| Gruber (2005) | 1 | 1 | 0 | 0 | 1 | 0 | 0 | 1 | 0 | 1 | 0 | 1 |  |  | 0 | ***0.5*** |

*Note.* 1 = criterion was endorsed, 2 = criterion was not endorsed, _ = not applicable; *Criterion I (Objective) =* was the research question or objective in this paper clearly stated?; *Criterion II* (Population) = Was the study population clearly specified and defined?; *Criterion III* (Partic. rate) = Was the participation rate of eligible persons at least 50%?; *Criterion IVa* (Same pop/period) = Were all the subjects selected or recruited from the same or similar populations (including the same time period)? *Criterion IVb* (Uniform inc/ex)= Were inclusion and exclusion criteria for being in the study prespecified and applied uniformly to all participants?; *Criterion V (Power) =* Was a sample size justification, power description, or variance and effect estimates provided?; *Criterion VI* (I.v. prior d.v.) = For the analyses in this paper, were the exposure(s) of interest measured prior to the outcome(s) being measured?; *Criterion VII* (Time i.v. & d.v.) = Was the timeframe sufficient so that one could reasonably expect to see an association between exposure and outcome if it existed?; *Criterion VIII (Corr/subgroup) =* For exposures that can vary in amount or level, did the study examine different levels of the exposure as related to the outcome (e.g., categories of exposure, or exposure measured as continuous variable)?; *Criterion IX (I.v. defined) =* Were the exposure measures (independent variables) clearly defined, valid, reliable, and implemented consistently across all study participants?; *Criterion X (I.v. dep. meas) =* Was the exposure(s) assessed more than once over time?; *Criterion XI (D.v. defined) =* Were the outcome measures (dependent variables) clearly defined, valid, reliable, and implemented consistently across all study participants?; *Criterion XII (Blind) =* Were the outcome assessors blinded to the exposure status of participants?; *Criterion XIII (Loss 20% FU) =* Was loss to follow-up after baseline 20% or less?; *Criterion XIV (Confounder) =* Were key potential confounding variables measured and adjusted statistically for their impact on the relationship between exposure(s) and outcome(s)?

## Criteria against which the literature rated was rated as high quality and low risk of bias

All studies were rated to have a low risk of bias against 9 of the 14 criteria of the risk of bias assessment (i.e., criteria I, II, IVa, IVb, VII, VIII, IX, XI, and XIV). First, all studies *clearly stated* their *research question or objective* (criterion I), *clearly specified and defined study populations* as they included cannabis users and controls as defined by each study (criterion II), and *uniformly applied prespecified inclusion and exclusion criteria* (criterion IVb). Second, criterion IVa refers to the *selection or recruitment of all participants from the same or similar populations, including the same time period*. Indeed, overlapping (or partly overlapping) datasets were used by 7 studies. Of these, 2 studies examined data extracted from the same dataset (Epstein et al., 2014; Epstein and Kumra, 2015).

Four studies included cannabis users and controls using different recruitment strategies: cannabis users from programs for chemical dependency, and controls from similar geographic areas (Epstein et al., 2014; Epstein and Kumra, 2015); cannabis users from enrolments in a residential drug-rehabilitation centre, and controls from an adolescent medical clinic (Ashtari et al., 2009); and cannabis users and controls from different studies (Becker et al., 2015). Participants recruitment was unclear in 3 studies (Gruber and Yurgelun-Todd, 2005; Yucel et al., 2016; Levar et al., 2018).

Third, the timeframe used by the reviewed studies was considered *sufficient so that one could reasonably expect to see an association between* cannabis *exposure and* white matter *outcome if it existed* (criterion 7). Longitudinal studies used sufficient timeframes to detect changes in brain integrity (e.g., 18-months, 2-years and 3-years (Jacobus et al., 2013a; Jacobus et al., 2013b; Becker et al., 2015; Epstein and Kumra, 2015; Lichenstein et al., 2022)). Cross-sectional studies included participants with regular cannabis use using heterogeneous criteria outlined in Supplementary Table 2.

Fourth, in the reviewed studies *exposure measures (independent variables)* and *outcome measures (dependent variables) were clearly defined, valid, reliable, and implemented consistently across all study participants* (criteria IX and XI). Specifically, they defined the required criteria for cannabis use and by measuring cannabis exposure metrics in their samples, and robustly measured the outcome variables using reliable diffusion-weighted imaging data acquisition and analyses methods and techniques.

Fifth, *loss at follow-up* (criterion XIII) was reported by all of the 5 longitudinal studies reviewed, and amounted to ~20% (Becker et al., 2015; Epstein and Kumra, 2015) and < 20% (Jacobus et al., 2013a; Jacobus et al., 2013b; Lichenstein et al., 2022). The loss at follow up was not applicable for the remainder cross-sectional studies. Most studies specified the *timeframe between exposure and outcome* (i.e., criterion VII, ranked as 0.8 out of 1). Indeed, most studies reported the duration of cannabis use (i.e., exposure) before testing brain integrity (i.e., outcome) - or provided information on the age of cannabis use onset that was used to index duration of cannabis use*.* Further, longitudinal studies monitored cannabis use between baseline and follow-up (Jacobus et al., 2013a; Jacobus et al., 2013b; Becker et al., 2015; Epstein and Kumra, 2015; Lichenstein et al., 2022).

Lastly, the literature was rated highly for the *measurement* of *key potential confounding variables and* their *statistical adjustment for their impact on the relationship between* cannabis *exposure and* white matter integrity *outcomes* (criterion XIV). Twenty three of 30 studies controlled for at least 1 confounding variable when analyzing the impact of cannabis exposure on white matter. Studies varied widely on which variables were considered confounders (Supplementary Figure 4). While 5 studies did not statistically adjust for confounding variables, they reported non-significant group differences for such variables e.g., age, sex IQ, alcohol and tobacco use, socio-economic status, and handedness (Gruber and Yurgelun-Todd, 2005; Yücel et al., 2010; Gruber et al., 2011; Gruber et al., 2014; Manza et al., 2020). A single study accounted for age and alcohol consumption in brain-cannabis exposure correlations but not in the group comparison (Arnone et al., 2008).

 **Supplementary Figure 4: Overview of how the literature measured and accounted for confounding variables**

*Note.* Bava and colleagues (2009; 2010) reported on the same sample, so each confounding variable is counted only once here. SUD = Substance Use Disorder

## Criteria against which the literature had ratings of moderate quality and risk of bias

The literature was rated moderately biased for 1 criterion (i.e., 0.6 out of 1). Criterion VIII was rated as having a moderate quality rating (i.e., 0.6). Specifically, the reviewed literature included *exposures that can vary in amount or level* (i.e., such as cannabis exposure); and only some of the reviewed studies *examined different levels of exposure as related to the outcome.* Specifically, 13 studies used cannabis *exposure measured as a continuous* variable and examined it in relation to brain integrity via correlational analyses. Further, 2 other studies examined how *categories of exposure affected brain integrity via* running *subgroup analysis.* 1 study separated cannabis users into low versus high potency users, daily versus occasional users, and age at first use (before 15 years of age compared to after 15 years of age) (Rigucci et al., 2016), while another study evaluated differences in FA in early versus late onset cannabis users (with the onset of regular use before or after 16 years of age) (Gruber et al., 2014).

## Criteria against which the literature was rated as low quality and high risk of bias

The literature was rated as 0 out of 1 against several criteria (i.e., criteria III, V, VI, X). First, all studies were cross-sectional, except for longitudinal studies that assessed cannabis *exposure more than once over time* (criterion X, rated 0.2 out of 1). Second, no study reported if the *participation rate of eligible persons was at least 50%* from the participant sample pool (criterion III). Third, only 2 studies *described power analyses* or justified the inclusion of the sample size (criterion V) (Manza et al., 2020; Knodt et al., 2022) and could have led to insufficiently powered samples to detect significant group differences (Ashtari et al., 2009; Epstein et al., 2014; Epstein and Kumra, 2015; Levar et al., 2018; Cousijn et al., 2022), or could have limited the detection of differences with small effect sizes (Jakabek et al., 2016; Cousijn et al., 2022). 1 study conducted a power analysis for the conduct of a statistical analysis i.e., analysis of covariance analysis (Yucel et al., 2016). Further, the *exposure(s) of interest* (i.e., cannabis use) were not *prior to the outcomes* measured (i.e., brain integrity), in none of the reviewed studies, as per criterion VI. Indeed, cannabis exposure was assessed at the same testing session where brain integrity was, even in longitudinal studies as at baseline cannabis use was already present.

Finally, rating of the literature against criterion XII was not applicable as *the outcome assessors* could not be blinded *to* cannabis *exposure status of participants* in order to conduct testing, and this was not required given the lack of observational nature of the studies. Further, robust neuroimaging tools have been shown to be robust and replicable, therefore mitigating the risk of bias. Interestingly, 1 study reported that a blinded rater checked scans for motion and other artifacts, to additionally ensure high data integrity (Epstein and Kumra, 2015).

# References

Arnone, D., Barrick, T.R., Chengappa, S., Mackay, C.E., Clark, C.A., and Abou-Saleh, M.T. (2008). Corpus callosum damage in heavy marijuana use: Preliminary evidence from diffusion tensor tractography and tract-based spatial statistics. *NeuroImage* 41(3)**,** 1067-1074. doi: 10.1016/j.neuroimage.2008.02.064.

Ashtari, M., Cervellione, K., Cottone, J., Ardekani, B.A., and Kumra, S. (2009). Diffusion abnormalities in adolescents and young adults with a history of heavy cannabis use. *J Psychiatr Res* 43(3)**,** 189-204. doi: 10.1016/j.jpsychires.2008.12.002.

Bava, S., Frank, L.R., McQueeny, T., Schweinsburg, B.C., Schweinsburg, A.D., and Tapert, S.F. (2009). Altered white matter microstructure in adolescent substance users. *Psychiatry Res Neuroimaging* 173(3)**,** 228-237. doi: 10.1016/j.pscychresns.2009.04.005.

Bava, S., Jacobus, J., Mahmood, O., Yang, T.T., and Tapert, S.F. (2010). Neurocognitive correlates of white matter quality in adolescent substance users. *Brain Cogn* 72(3)**,** 347-354. doi: 10.1016/j.bandc.2009.10.012.

Becker, M.P., Collins, P.F., Lim, K.O., Muetzel, R.L., and Luciana, M. (2015). Longitudinal changes in white matter microstructure after heavy cannabis use. *Dev Cogn Neurosci* 16**,** 23-35. doi: 10.1016/j.dcn.2015.10.004.

Cousijn, J., Toenders, Y.J., van Velzen, L.S., and Kaag, A.M. (2022). The relation between cannabis use, dependence severity and white matter microstructure: A diffusion tensor imaging study. *Addict Biol* 27(1)**,** e13081. doi: 10.1111/adb.13081.

Delisi, L.E., Bertisch, H.C., Szulc, K.U., Majcher, M., Brown, K., Bappal, A., et al. (2006). A preliminary DTI study showing no brain structural change associated with adolescent cannabis use. *Harm Reduction Journal* 3. doi: 10.1186/1477-7517-3-17.

Epstein, K.A., Cullen, K.R., Mueller, B.A., Robinson, P., Lee, S., and Kumra, S. (2014). White Matter Abnormalities and Cognitive Impairment in Early-Onset Schizophrenia-Spectrum Disorders. *J Am Acad Child Adolesc Psychiatry* 53(3)**,** 362-372. doi: 10.1016/j.jaac.2013.12.007.

Epstein, K.A., and Kumra, S. (2015). White matter fractional anisotropy over two time points in early onset schizophrenia and adolescent cannabis use disorder: A naturalistic diffusion tensor imaging study. *Psychiatry Res Neuroimaging* 232(1)**,** 34-41. doi: 10.1016/j.pscychresns.2014.10.010.

Filbey, F.M., Aslan, S., Calhoun, V.D., Spence, J.S., Damaraju, E., Caprihan, A., et al. (2014). Long-term effects of marijuana use on the brain. *Proc Natl Acad Sci U.S.A* 111(47)**,** 16913-16918. doi: 10.1073/pnas.1415297111.

Gruber, S.A., Dahlgren, M.K., Sagar, K.A., Gonenc, A., and Lukas, S.E. (2014). Worth the wait: Effects of age of onset of marijuana use on white matter and impulsivity. *Psychopharmacology* 231(8)**,** 1455-1465. doi: 10.1007/s00213-013-3326-z.

Gruber, S.A., Silveri, M.M., Dahlgren, M.K., and Yurgelun-Todd, D. (2011). Why So Impulsive? White Matter Alterations Are Associated With Impulsivity in Chronic Marijuana Smokers. *Exp Clin Psychopharmacol* 19(3)**,** 231-242. doi: 10.1037/a0023034.

Gruber, S.A., and Yurgelun-Todd, D.A. (2005). Neuroimaging of marijuana smokers during inhibitory processing: A pilot investigation. *Cogn Brain Res* 23(1)**,** 107-118. doi: 10.1016/j.cogbrainres.2005.02.016.

Jacobus, J., McQueeny, T., Bava, S., Schweinsburg, B.C., Frank, L.R., Yang, T.T., et al. (2009). White matter integrity in adolescents with histories of marijuana use and binge drinking. *Neurotoxicol Teratol* 31(6)**,** 349-355. doi: 10.1016/j.ntt.2009.07.006.

Jacobus, J., Squeglia, L.M., Bava, S., and Tapert, S.F. (2013a). White matter characterization of adolescent binge drinking with and without co-occurring marijuana use: A 3-year investigation. *Psychiatry Res Neuroimaging* 214(3)**,** 374-381. doi: 10.1016/j.pscychresns.2013.07.014.

Jacobus, J., Thayer, R.E., Trim, R.S., Bava, S., Frank, L.R., and Tapert, S.F. (2013b). White Matter Integrity, Substance Use, and Risk Taking in Adolescence. *Psychol Addict Behav* 27(2)**,** 431-442. doi: 10.1037/a0028235.

Jakabek, D., Yücel, M., Lorenzetti, V., and Solowij, N. (2016). An MRI study of white matter tract integrity in regular cannabis users: Effects of cannabis use and age. *Psychopharmacology* 233(19-20)**,** 3627-3637. doi: 10.1007/s00213-016-4398-3.

Kim, D.-J., Skosnik, P.D., Cheng, H., Pruce, B.J., Brumbaugh, M.S., Vollmer, J.M., et al. (2011). Structural network topology revealed by white matter tractography in cannabis users: A graph theoretical analysis. *Brain Connect* 1(6)**,** 473-483. doi: 10.1089/brain.2011.0053.

Knodt, A.R., Meier, M.H., Ambler, A., Gehred, M.Z., Harrington, H., Ireland, D., et al. (2022). Diminished Structural Brain Integrity in Long-term Cannabis Users Reflects a History of Polysubstance Use. *Biol Psychiatry* 92(11)**,** 861-870. doi: 10.1016/j.biopsych.2022.06.018.

Koenis, M.M.G., Durnez, J., Rodrigue, A.L., Mathias, S.R., Alexander-Bloch, A.F., Barrett, J.A., et al. (2021). Associations of cannabis use disorder with cognition, brain structure, and brain function in African Americans. *Hum Brain Mapp* 42(6)**,** 1727-1741. doi: 10.1002/hbm.25324.

Levar, N., Francis, A.N., Smith, M.J., Ho, W.C., and Gilman, J.M. (2018). Verbal Memory Performance and Reduced Cortical Thickness of Brain Regions Along the Uncinate Fasciculus in Young Adult Cannabis Users. *Cannabis Cannabinoid Res* 3(1)**,** 56-65. doi: 10.1089/can.2017.0030.

Lichenstein, S.D., Shaw, D.S., and Forbes, E.E. (2022). Cannabis, connectivity, and coming of age: Associations between cannabis use and anterior cingulate cortex connectivity during the transition to adulthood. *Front Hum Neurosci* 16**,** 951204. doi: 10.3389/fnhum.2022.951204.

Lorenzetti, V., Solowij, N., and Yucel, M. (2016). The Role of Cannabinoids in Neuroanatomic Alterations in Cannabis Users. *Biol Psychiatry* 79(7)**,** e17-31. doi: 10.1016/j.biopsych.2015.11.013.

Manza, P., Yuan, K., Shokri-Kojori, E., Tomasi, D., and Volkow, N.D. (2020). Brain structural changes in cannabis dependence: Association with MAGL. *Mol Psychiatry* 25(12)**,** 3256-3266. doi: 10.1038/s41380-019-0577-z.

Orr, J.M., Paschall, C.J., and Banich, M.T. (2016). Recreational marijuana use impacts white matter integrity and subcortical (but not cortical) morphometry. *NeuroImage Clin* 12**,** 47-56. doi: 10.1016/j.nicl.2016.06.006.

Rigucci, S., Marques, T.R., Di Forti, M., Taylor, H., Dell'Acqua, F., Mondelli, V., et al. (2016). Effect of high-potency cannabis on corpus callosum microstructure. *Psychol Med* 46(4)**,** 841-854. doi: 10.1017/s0033291715002342.

Shollenbarger, S.G., Price, J., Wieser, J., and Lisdahl, K. (2015). Poorer frontolimbic white matter integrity is associated with chronic cannabis use, FAAH genotype, and increased depressive and apathy symptoms in adolescents and young adults. *NeuroImage Clin* 8**,** 117-125. doi: 10.1016/j.nicl.2015.03.024.

Sweigert, J., Pagulayan, K., Greco, G., Blake, M., Larimer, M., and Kleinhans, N.M. (2020). A multimodal investigation of cerebellar integrity associated with high-risk cannabis use. *Addict Biol* 25(6). doi: 10.1111/adb.12839.

Ugurbil, K., Xu, J., Auerbach, E.J., Moeller, S., Vu, A.T., Duarte-Carvajalino, J.M., et al. (2013). Pushing spatial and temporal resolution for functional and diffusion MRI in the Human Connectome Project. *NeuroImage* 80**,** 80-104. doi: 10.1016/j.neuroimage.2013.05.012.

Yucel, M., Lorenzetti, V., Suo, C., Zalesky, A., Fornito, A., Takagi, M.J., et al. (2016). Hippocampal harms, protection and recovery following regular cannabis use. *Transl Psychiatry* 6. doi: 10.1038/tp.2015.201.

Yücel, M., Zalesky, A., Takagi, M.J., Bora, E., Fornito, A., Ditchfield, M., et al. (2010). White-matter abnormalities in adolescents with long-term inhalant and cannabis use: A diffusion magnetic resonance imaging study. *J Psychiatry Neurosci* 35(6)**,** 409-412. doi: 10.1503/jpn.090177.

Zalesky, A., Solowij, N., Yücel, M., Lubman, D.I., Takagi, M., Harding, I.H., et al. (2012). Effect of long-term cannabis use on axonal fibre connectivity. *Brain* 135(7)**,** 2245-2255. doi: 10.1093/brain/aws136.
